# Supplementary material for: Structural basis of peptide secretion for Quorum sensing by ComA
Source: Nat Commun. 2023 Nov 7;14:7178. doi: 10.1038/s41467-023-42852-9 (PMC10630487; doi:10.1038/s41467-023-42852-9)

Supplementary Materials for

Structure basis of peptide secretion for Quorum sensing by ComA

Lin Yu *et al.*

*Corresponding author. Email: lsham@nus.edu.sg and dbslmin@nus.edu.sg

This PDF file includes:

Supplementary Figures 1 to 18
Supplementary Tables 1 to 4
Source Data for Supplementary Figure 1

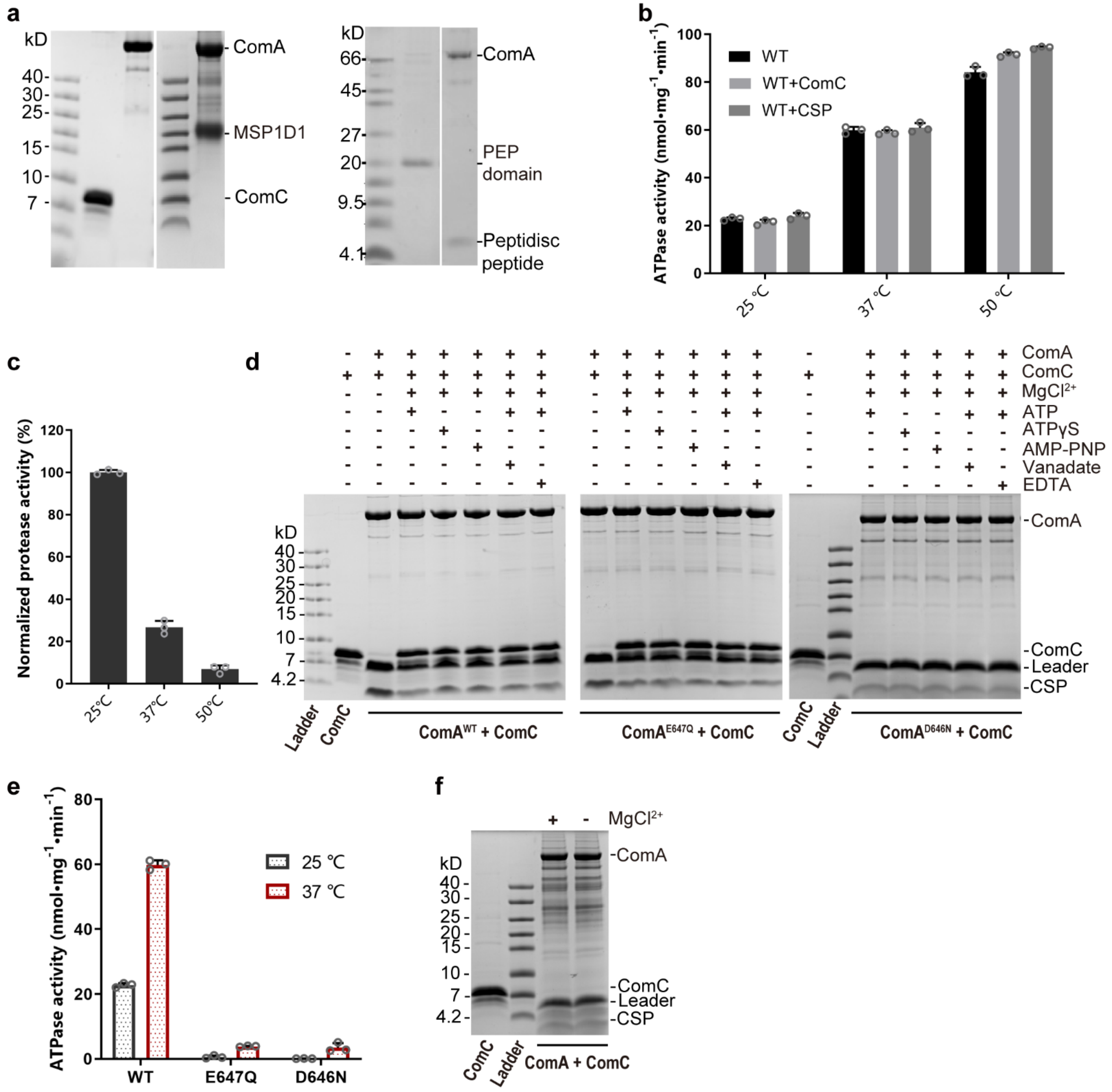

Supplementary Figure 1. Biochemical reconstitution and functional characterization of ComA. a, SDS-PAGE gel of purified ComC, ComA in detergents and nanodisc(left), truncated peptidase domain, and ComA reconstituted in peptidisc (right). **b,** ATPase and **c,** Peptidase activities of ComA under different reaction temperatures. **d,** The peptidase activity of both WT ComA (left) and E647Q mutant (middle) is inhibited by different nucleotides, while D646N mutant (right) is not. **e**, The ATPase activities of WT and ComA mutant (E647Q, D646N) under different reaction temperatures. **f,** Mg^{2+} alone has no effect on the peptidase activity of ComA. Individual data points are presented as circles whereas standard deviation (SD) are shown as error bars (n=3 for all samples.)

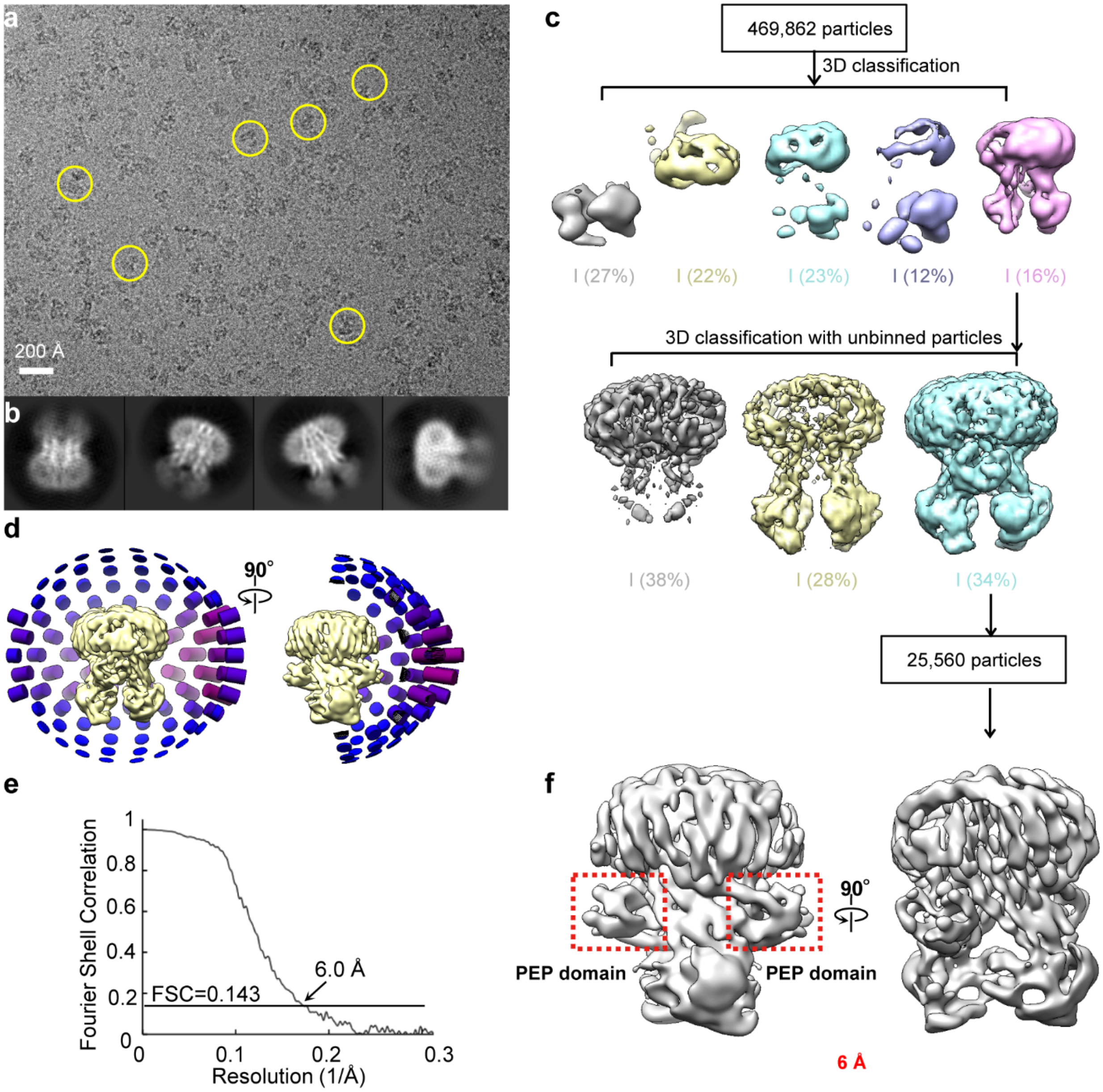

Supplementary Figure 2. Cryo-EM Single-Particle Analysis of ComA C17A in the presence of ComC. a, Display of a representative cryo-EM image, with distinct particles encircled for emphasis. **b,** The two-dimensional averages derived from the cryo-EM 2D-classification. The box dimension is established at 220 Å. **c,** A detailed flowchart outlining the image processing pathway. The final maps and their corresponding overall resolutions are highlighted in red. **d,** An illustrative representation of the angular distribution of cryo-EM particles included in the final 3D reconstruction. **e,** The Fourier Shell Correlation (FSC) curve showcasing the gold-standard FSC between two separate data maps. The indicated resolution at an FSC value of 0.143 is shown (FSC corrected applied). **f**, The final map of ComA C17A mutant presumably bound with ComC, with the PEP domain highlighted by a red dotted box.

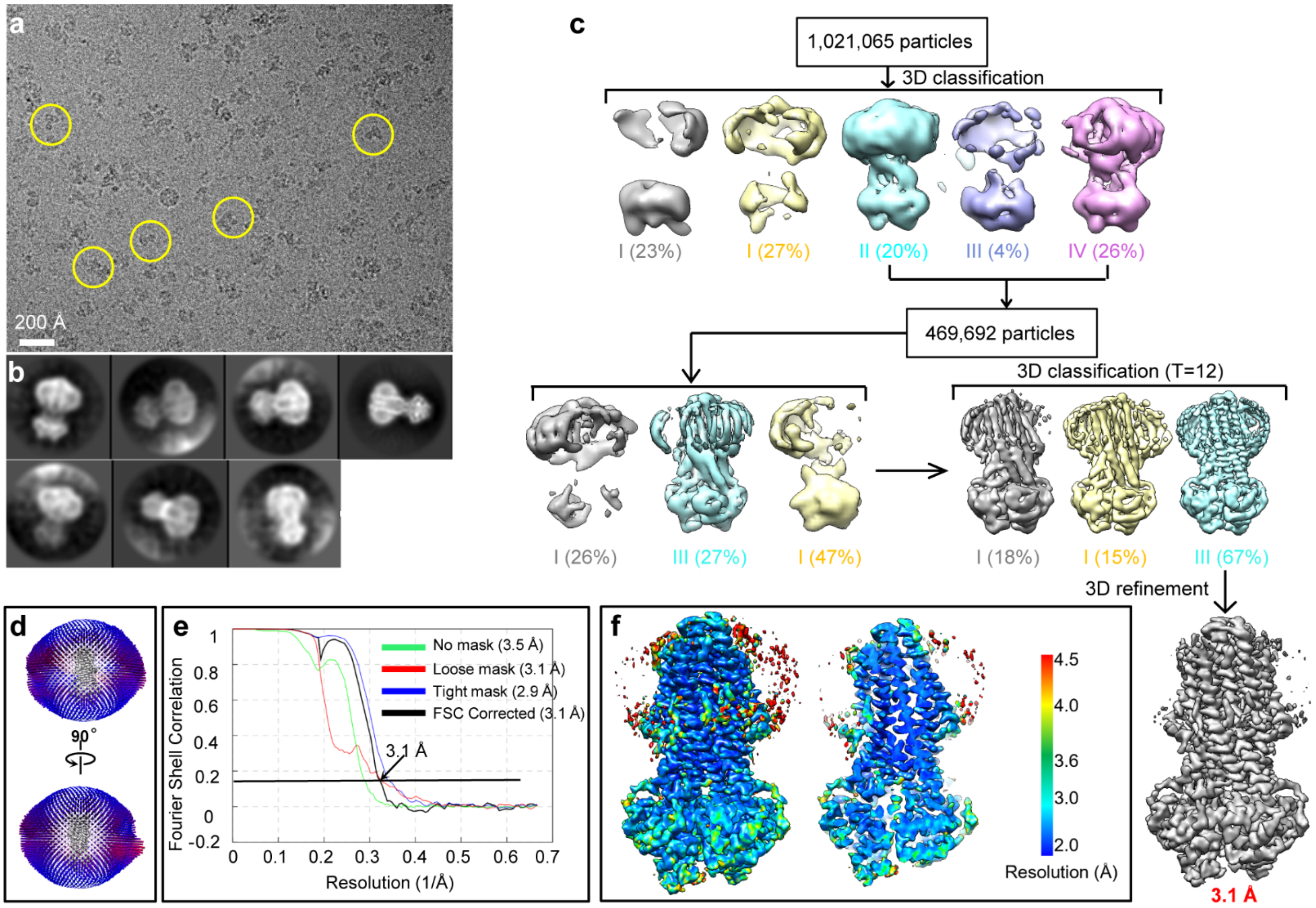

Supplementary Figure 3. Single-particle cryo-EM analysis of ATP-bound ComA (E647Q) in the absence of Mg^{2+}. **a,** Representative cryo-EM image with several particles marked by circles. **b,** 2D averages of cryo-EM particle images. The box dimension is 220 Å. **c,** Image processing flowchart. The final maps of one major conformation with its overall resolutions is indicated in red. **d,** The angular distribution of the cryo-EM particles included in the final 3D reconstruction. **e,** The Fourier shell correlation (FSC) curve: gold-standard FSC between two half data maps with indicated resolution at FSC=0.143 (FSC corrected applied); **f,** The surface and cross-sectional views of the cryo-EM maps filtered to the estimated overall resolution and colored according to local resolution.

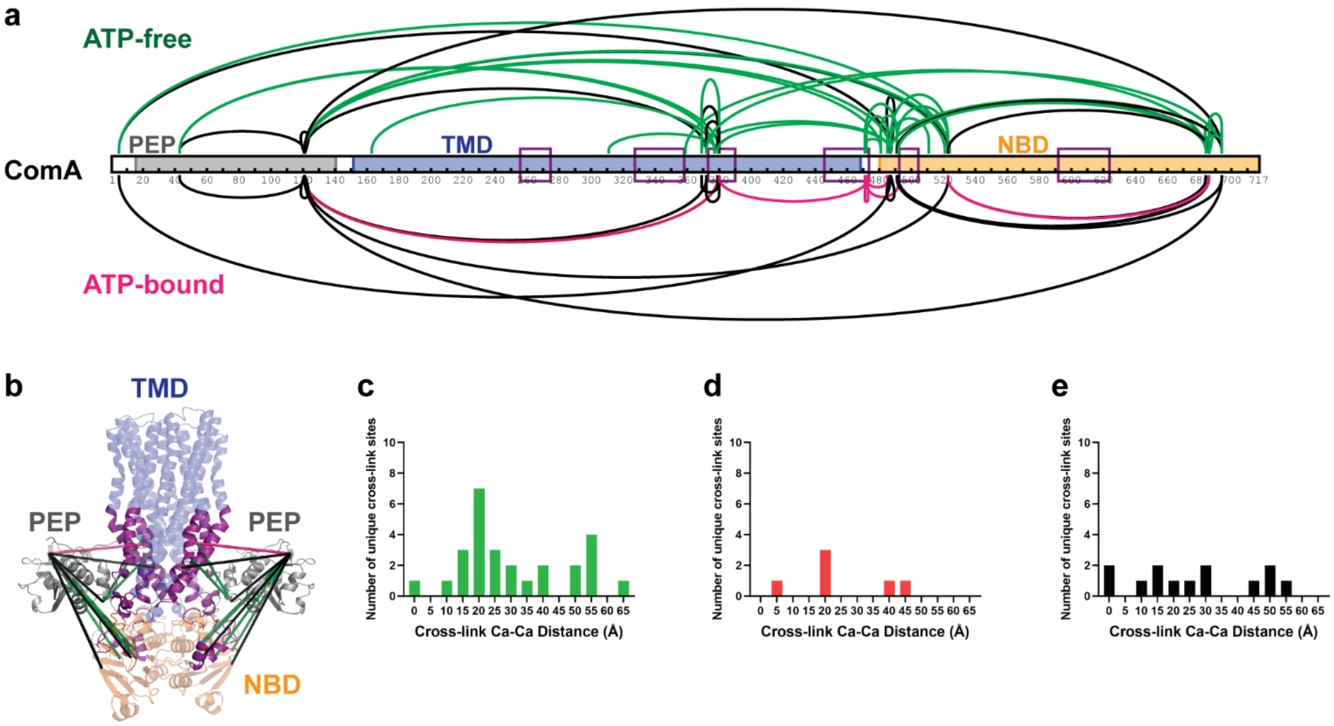

Supplementary Figure 4. Cross-linking Mass Spectrometry results of PEP location in the presence and absence of ATP. a, Cross-links from ATP-free (top) and ATP-bound ComA (bottom) are visualized across the ComA sequence. IC-gate residues are marked in purple boxes. B, Cross-links that fall on PEP region are mapped to cryo-EM structure of ComA**. c,d,e,** Histogram of distances for Cα-Cα distance for cross-links from ATP-free only, from ATP-bound only and for cross-links in both ATP-free and ATP-bound respectively. Cross-links are coloured green for ATP-free only, red for ATP-bound only and black for those found in both ATP-free and ATP-bound.

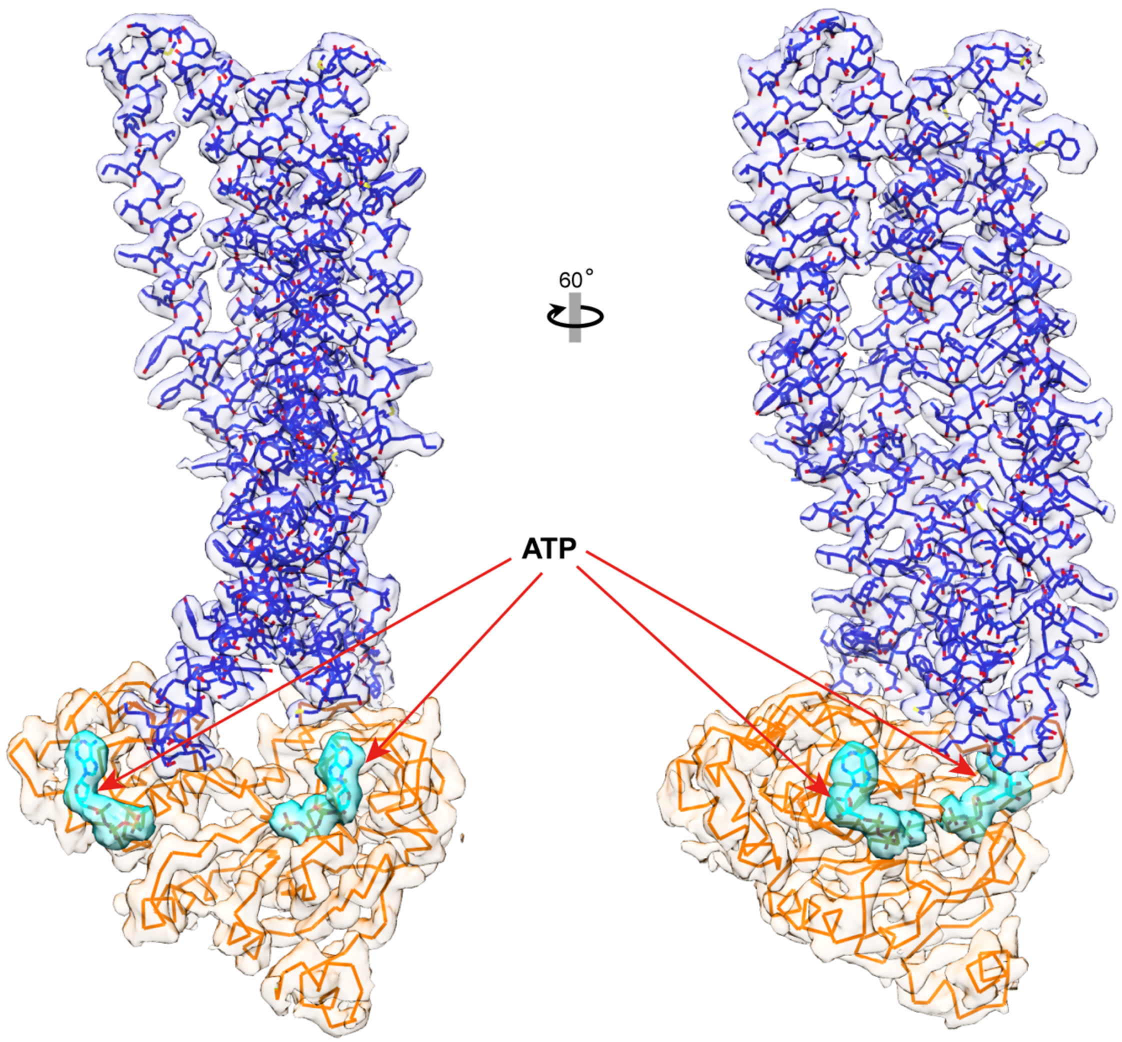

Supplementary Figure 5. Visualization of EM density of ComA-half in the ATP-bound map. The density of all side chains of TMD domain (colored in blue), NBD domain (colored in orange) and two bound ATP molecules (colored in cyan) are clearly resolved.

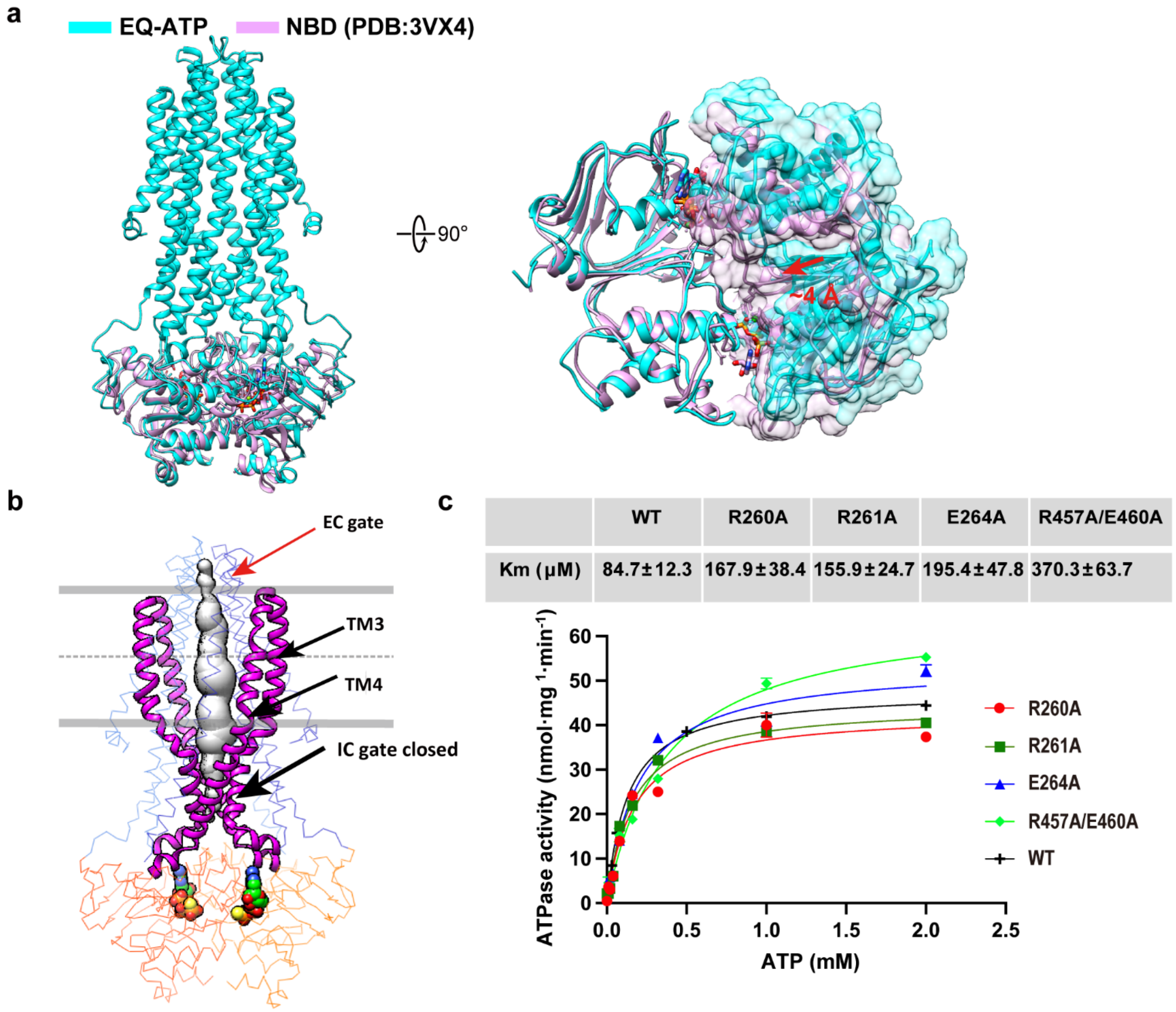

Supplementary Figure 6. Structural analysis of the first ComA (E647Q) structure captured with ATP-bound while Mg^{2+}-absent state. a, Structural comparison between ComA(E647Q)-ATP and the previously resolved ATP-Mg^{2+} -bound NBD domain alone structure (PDB ID:3VX4 [https://www.rcsb.org/structure/3VX4]). The RMSD is about 1.407 Å. ComA(E647Q)-ATP is depicted in cyan, and the NBD domain alone structure in pink. The NBD resolved in full-length ComA displays a less compact structure compared to the NBD domain alone structure. **b,** A ribbon-style representation of ATP-bound ComA, showcasing the central cavity in a grey surface representation. The color scheme includes TMD in blue, NBD in orange, IC gate-forming helices in magenta, and two bound ATP molecules shown as green spheres. ATP binding resulted in dimerized NBD domain while the two PEP domain become flexible. **c,** A depiction of the ATPase activity of ComA mutants located at the IC gate. The calculated Michaelis-Menten constants are provided at the top. The mutation of electrostatic residues in the IC gate region resulted in an increased Km value, suggesting a decreased ATP affinity. Individual data points are presented as circles whereas standard deviation (SD) are shown as error bars (n=3 for all samples.)

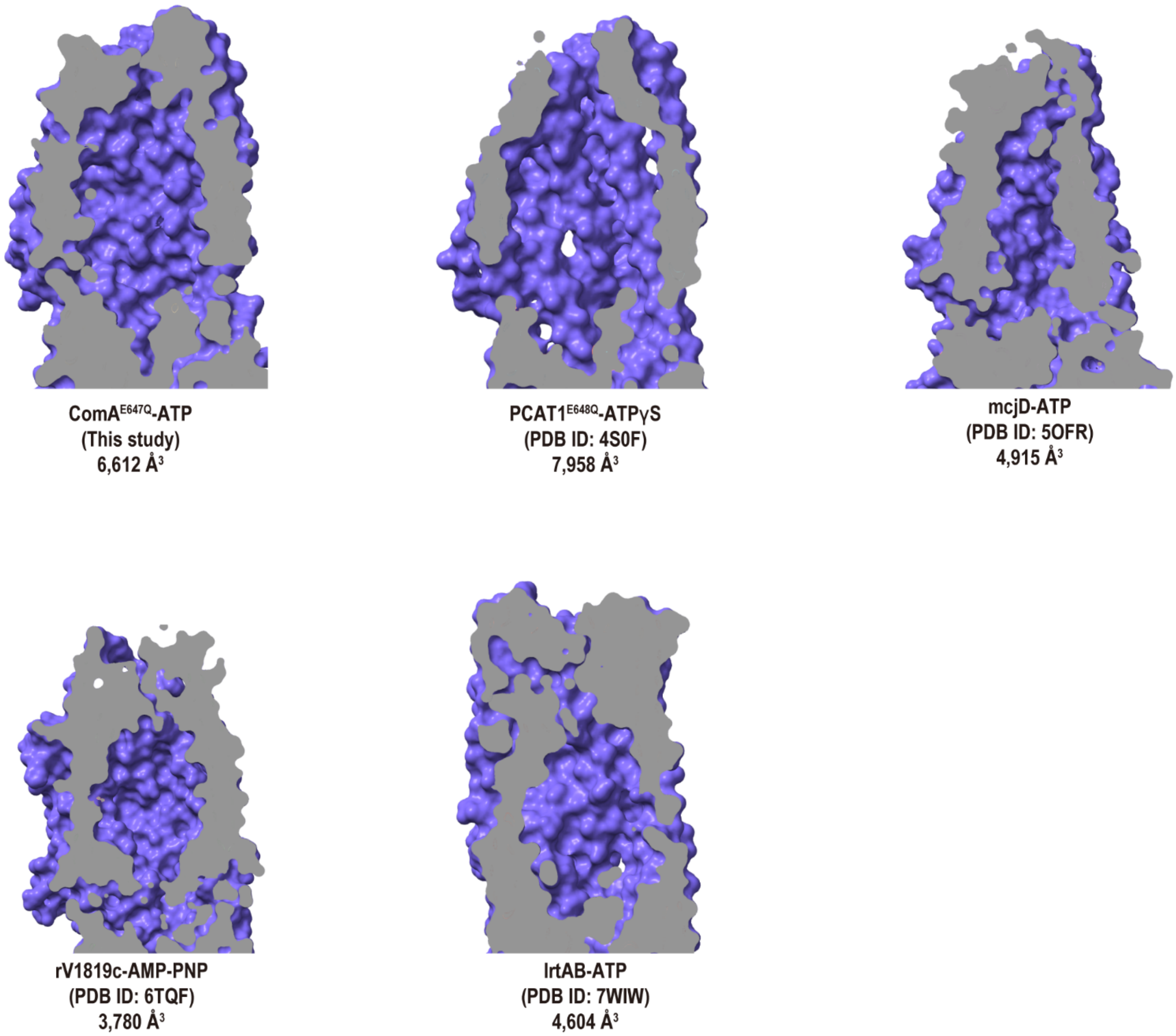

Supplementary Figure 7. Comparative Analysis of Central Cavity Size between ComA and Selected ABC Transporters.

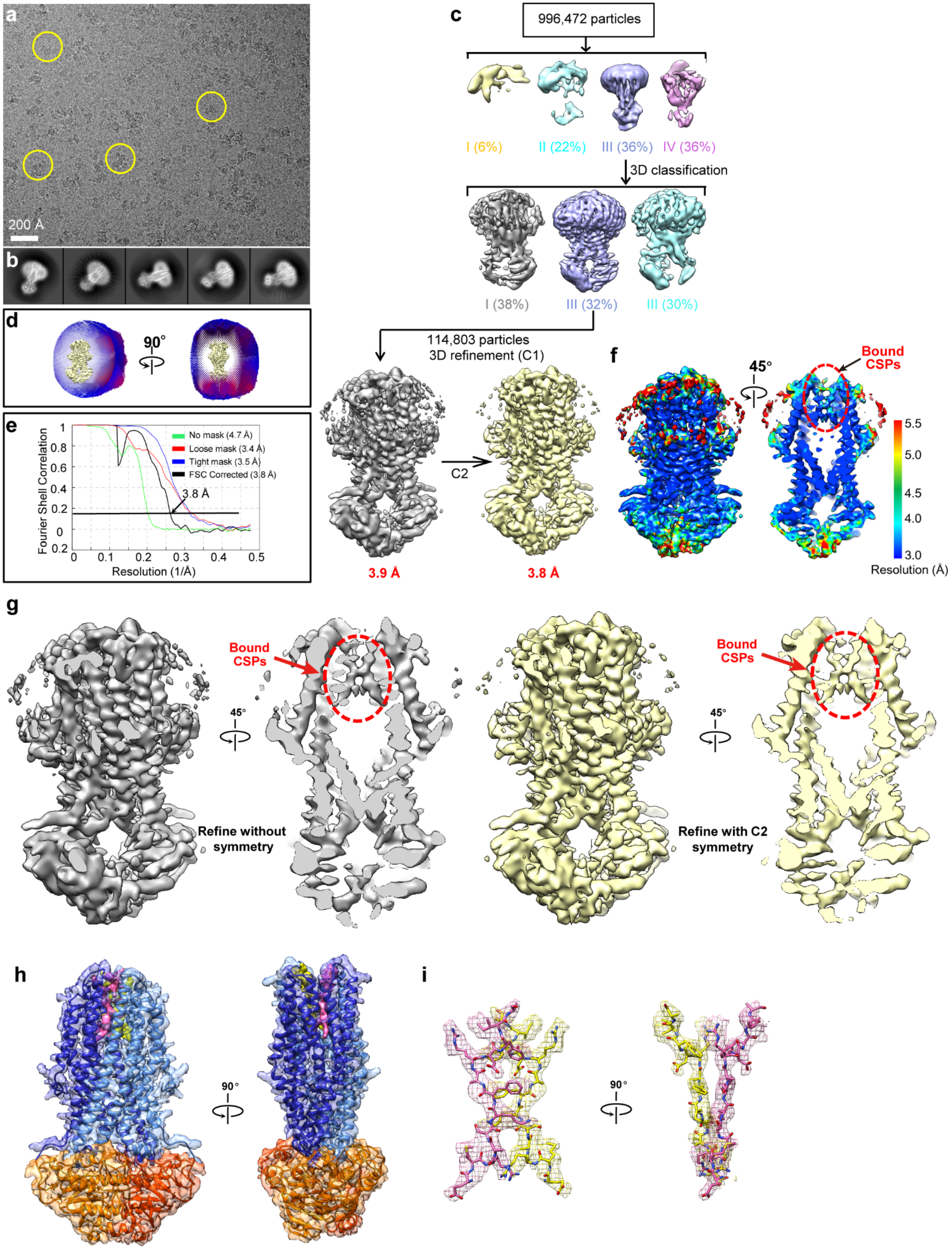
a
200 Å
b
c
996,472 particles
I (6%)
II (22%)
III (36%)
IV (36%)
3D classification
I (38%)
III (32%)
III (30%)
114,803 particles
3D refinement (C1)
C2
3.9 Å
3.8 Å
d
90°
e
Fourier Shell Correlation
Resolution (1/Å)
No mask (4.7 Å)
Loose mask (3.4 Å)
Tight mask (3.5 Å)
FSC Corrected (3.8 Å)
3.8 Å
f
45°
Bound CSPs
Resolution (Å)
5.5
5.0
4.5
4.0
3.0
g
Bound CSPs
45°
Refine without symmetry
Refine with C2 symmetry
h
90°
i
90°

Supplementary Figure 8. Single-particle cryo-EM analysis of CSP-bound ComA. a, Representative cryo-EM image with several particles marked by circles. **b,** 2D averages of cryo-EM particle images. The box dimension is 220 Å. **c,** Image processing flowchart. The final maps of one major conformation with its overall resolutions is indicated in red. **d,** The angular distribution of the cryo-EM particles included in the final 3D reconstruction. **e,** The Fourier shell correlation (FSC) curve: gold-standard FSC between two half data maps with indicated resolution at FSC=0.143 (FSC corrected applied); **f,** The surface, cross-sectional, and middle-layer views with two bound CSP molecules of the cryo-EM map filtered to the estimated overall resolution and colored according to local resolution. **g,** cryo-EM maps of CSP-bound ComA from 3D-refinement with and without symmetry. **h,** Superposition of cryo-EM density and the model for CSP-bound ComA. **i,** Superposition of cryo-EM density and the model for two potential CSP molecules. The contour level for EM density map shown in Chimera is 0.01.

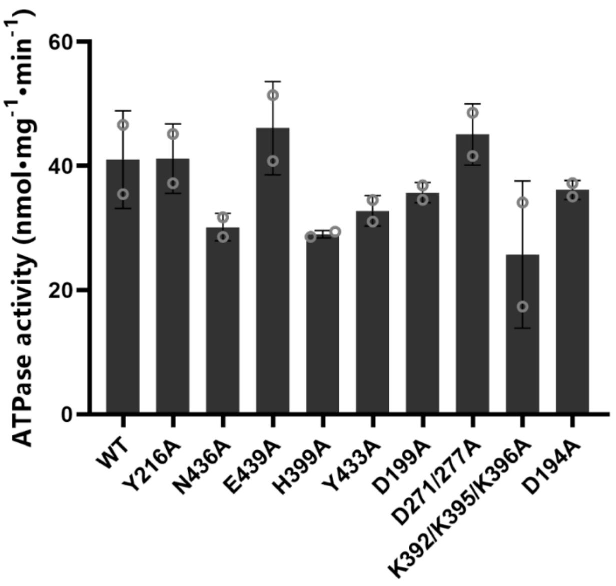

Supplementary Figure 9. ATPase activity of ComA mutants. Mutations of ComA at CSP-binding sites in the outer layer and charged residues located in the inner layer of the membrane have no significant effect on ATPase activity. Individual data points are presented as circles whereas standard deviation (SD) are shown as error bars (n=2 for all samples.)

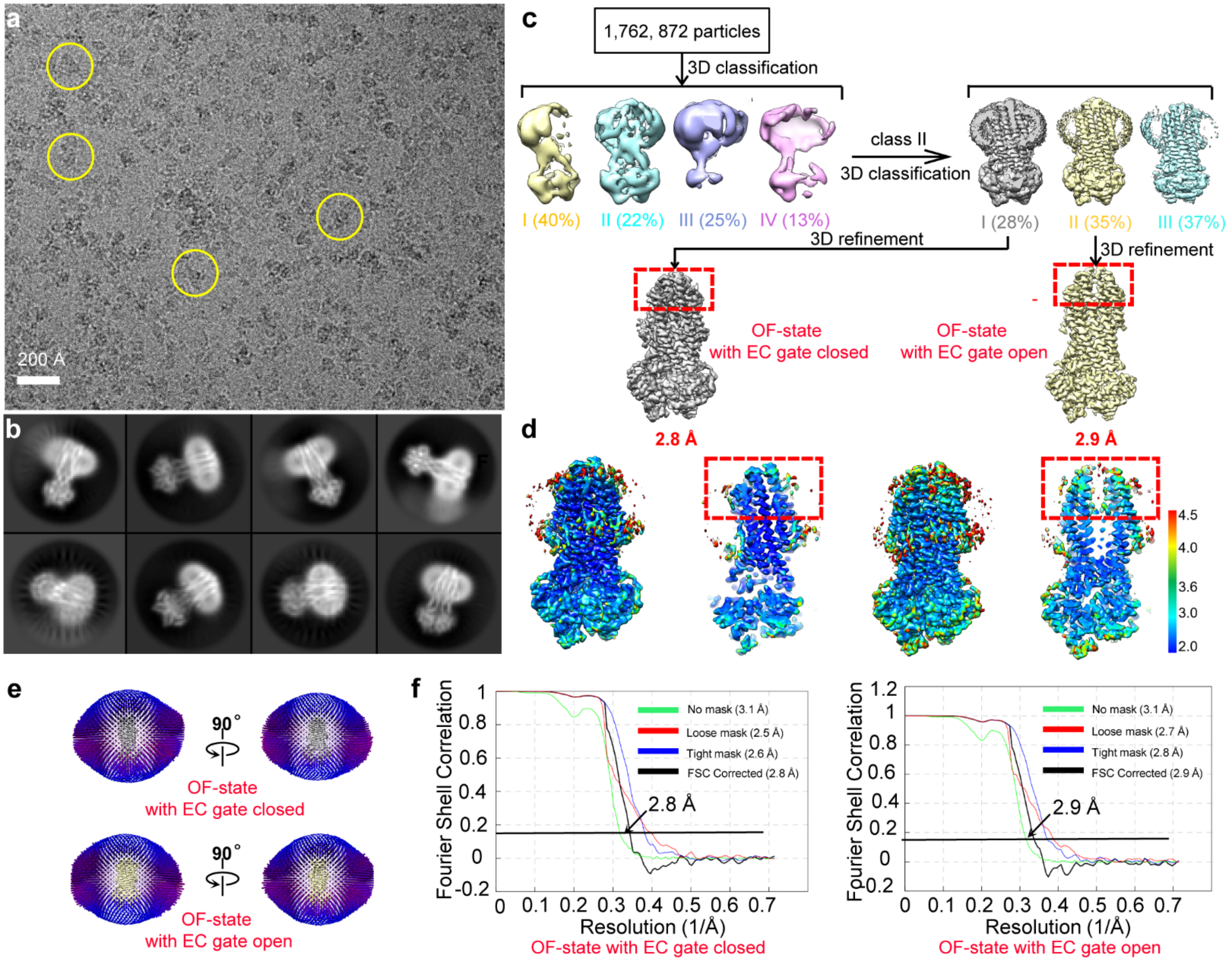

Supplementary Figure 10. Single-particle cryo-EM analysis of ATPγS-bound ComA in the presence of Mg^{2+}. a, Representative cryo-EM image with several particles marked by circles. **b,** 2D averages of cryo-EM particle images. The box dimension is 220 Å. **c,** Image processing flowchart. The final maps of two different conformations with their overall resolutions are indicated in red. **d,** The surface and cross-sectional views of the cryo-EM map filtered to the estimated overall resolution and colored according to local resolution. **e,** the angular distribution of the cryo-EM particles included in the final 3D reconstruction. **f,** The Fourier shell correlation (FSC) curve: gold-standard FSC between two half data maps with indicated resolution at FSC=0.143 (FSC corrected applied).

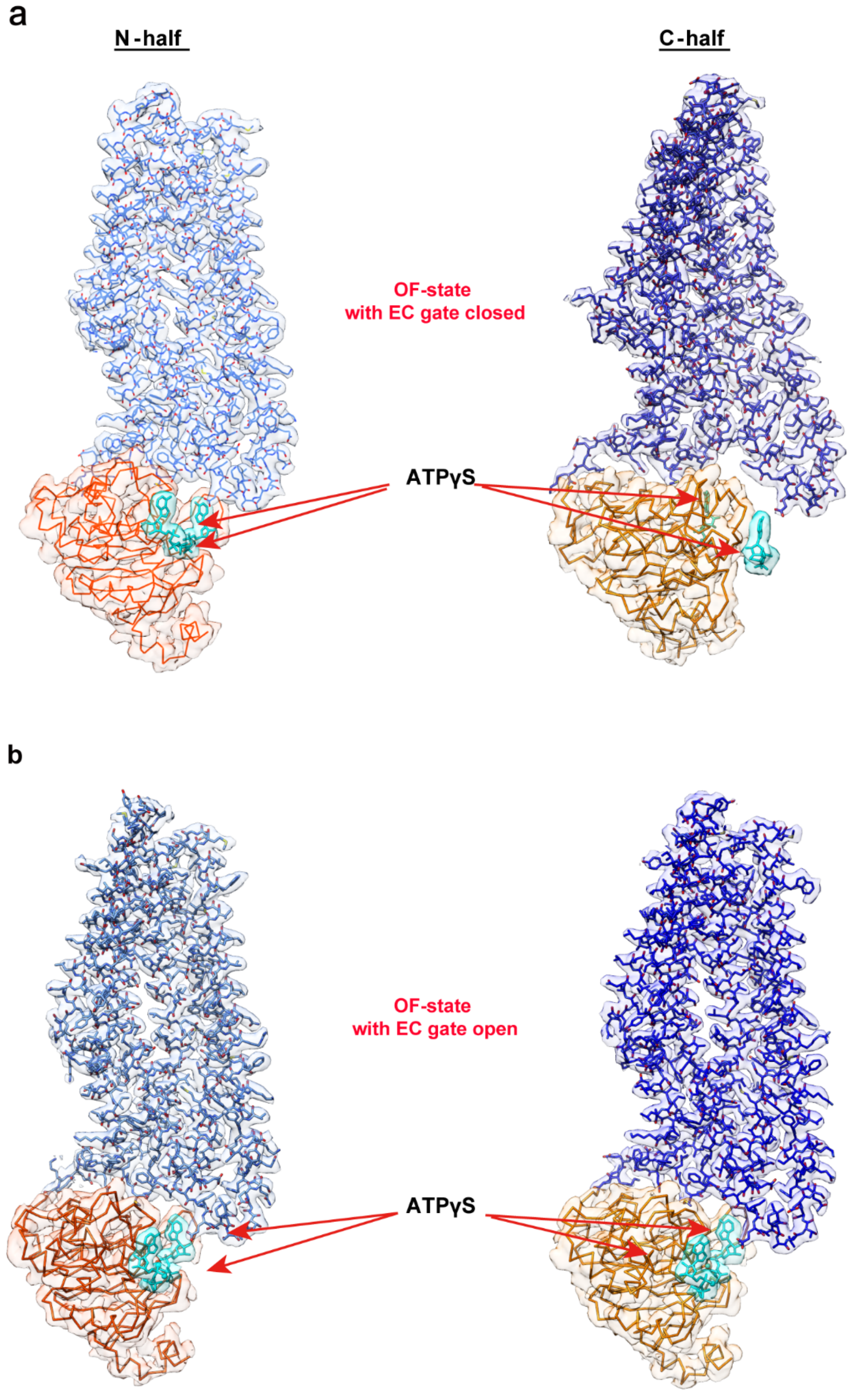

Supplementary Figure 11. Superposition of cryo-EM density and the model for ATPγS-bound ComA. a, OF-state with EC gate closed; **b,** OF-state with EC gate open. TMD is colored in blue, NBD is colored in orange, and bound ATPγS is colored in cyan.

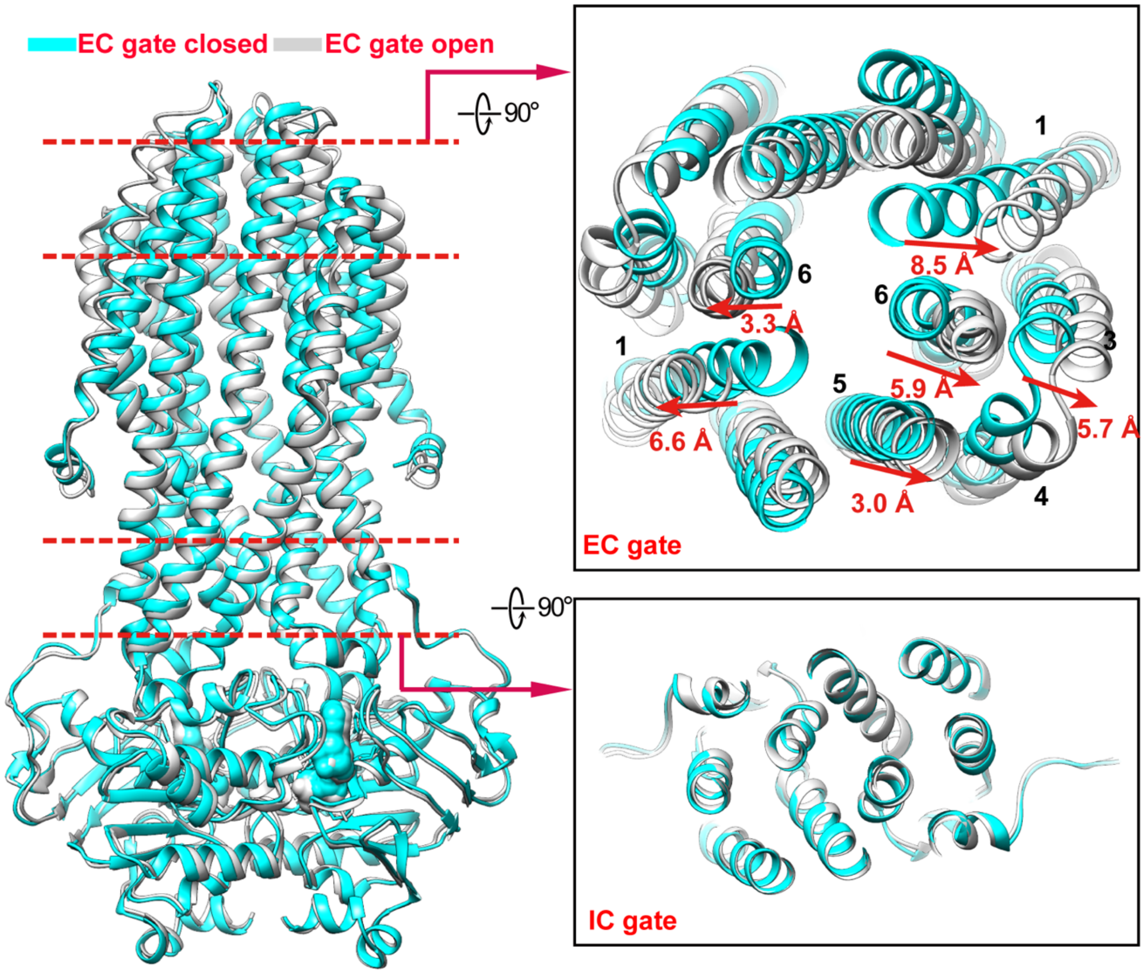

Supplementary Figure 12. Superposition of two distinct conformations with ATPγS bound. Left: OF-state with EC gate closed is in cyan, OF-state with EC gate open is in grey. Right upper: top-down view of EC gate; Right bottom: top-down views of IC gate for the superposition of two states

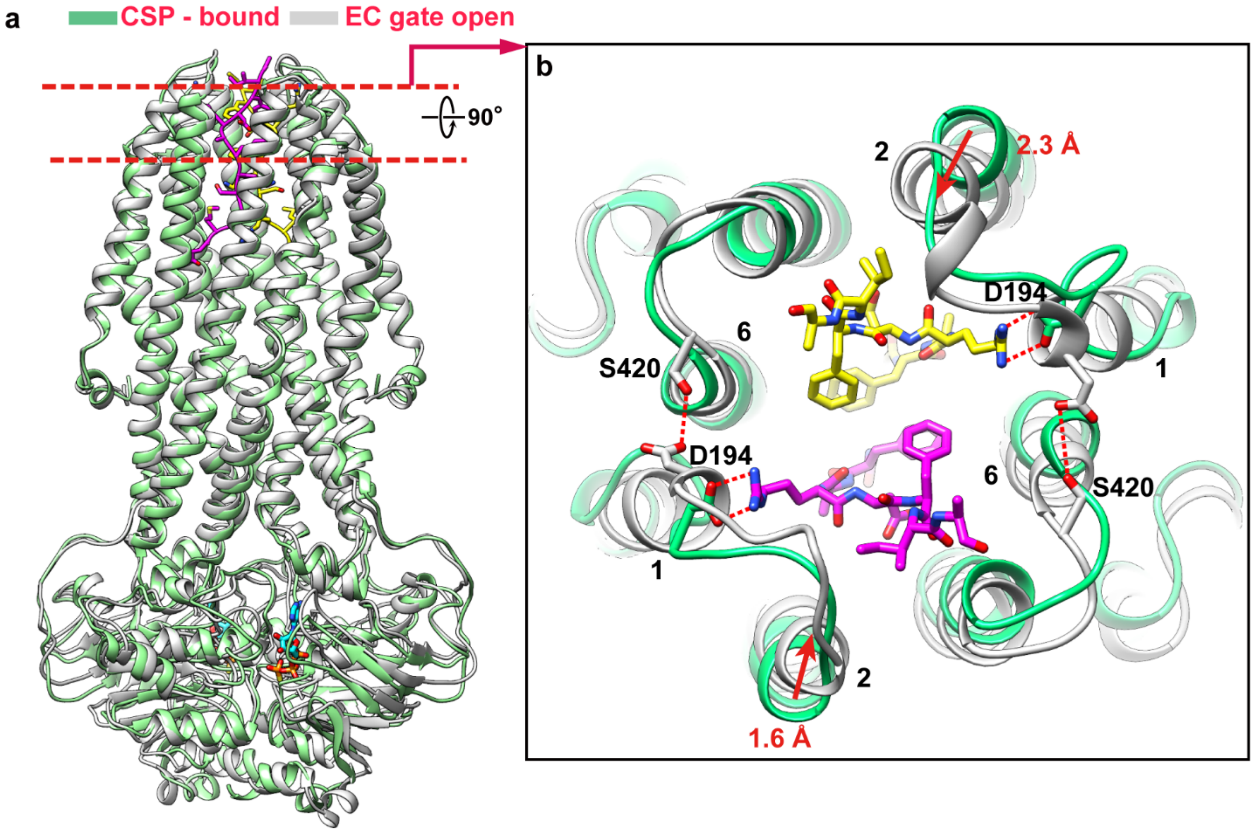

Supplementary Figure 13. Superposition of CSP-bound with OF-state with EC gate open ComA. a, Superposition of OF-open with EC gate open, and CSP bound structures. OF-open state is colored in grey, CSP bound state is colored in lime green. **b,** Top-down view of EC gate region with local rearrangements observed for CSP release.

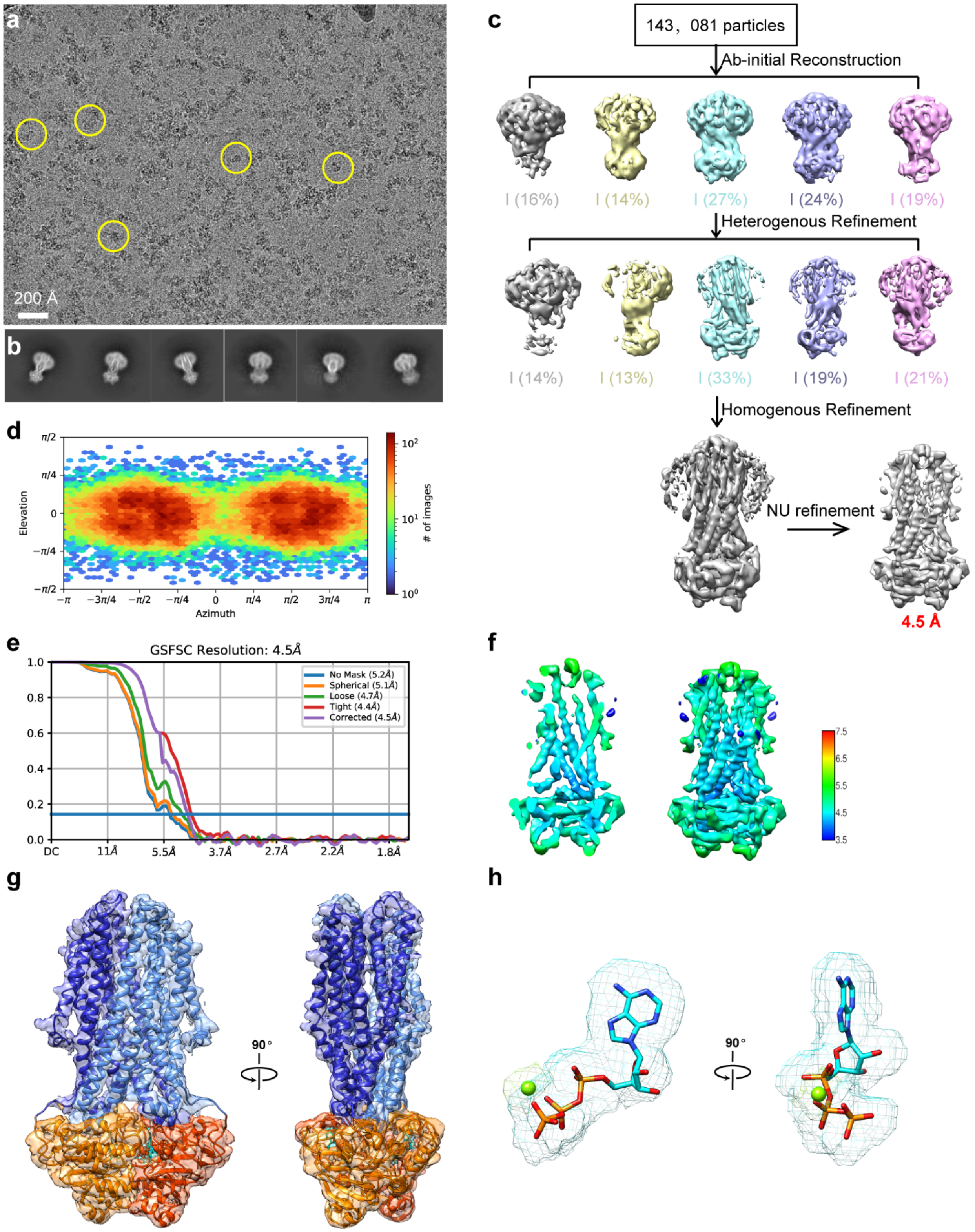

Supplementary Figure 14. Single-particle cryo-EM analysis of ATP-Mg^{2+} bound ComA (E647Q). a, Representative cryo-EM image with several particles marked by circles. **b,** 2D averages of cryo-EM particle images. The box dimension is 280 Å. **c,** Image processing flowchart. The final maps with overall resolutions are indicated in red. **d,** The angular distribution of the cryo-EM particles included in the final 3D reconstruction. **e,** The Fourier shell correlation (FSC) curve: gold standard FSC between two half data maps with indicated resolution at FSC=0.143 (FSC corrected applied). **f,** The surface cryo-EM map filtered to the estimated overall resolution and colored according to local resolution. **g,** Superposition of cryo-EM density and the model for ATP-Mg^{2+} bound ComA (E647Q). **h,** Superposition of cryo-EM density and the model for ATP and Mg^{2+} The contour level for EM density map in Chimera is 0.013.

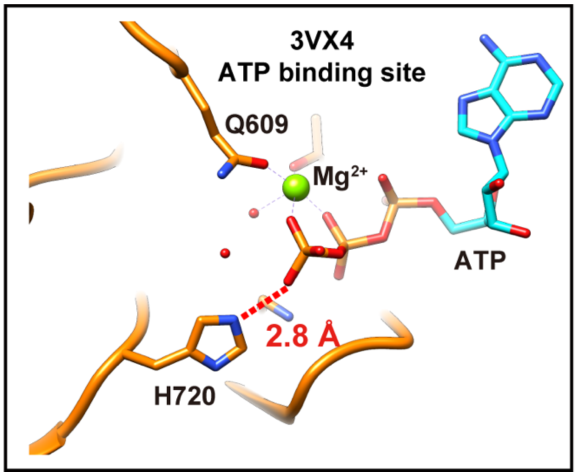

Supplementary Figure 15. Detailed structure of ATP binding site in the previously resolved ATP-Mg^{2+}-bound ComA NBD domain structure from *Strep. mutans* (PDB ID: 3VX4 [https://www.rcsb.org/structure/3VX4]). The residue His720, equivalent to His676 in *Strep. pneumoniae*, forms a salt bridge with the gamma phosphate when both ATP and Mg^{2+} are present.

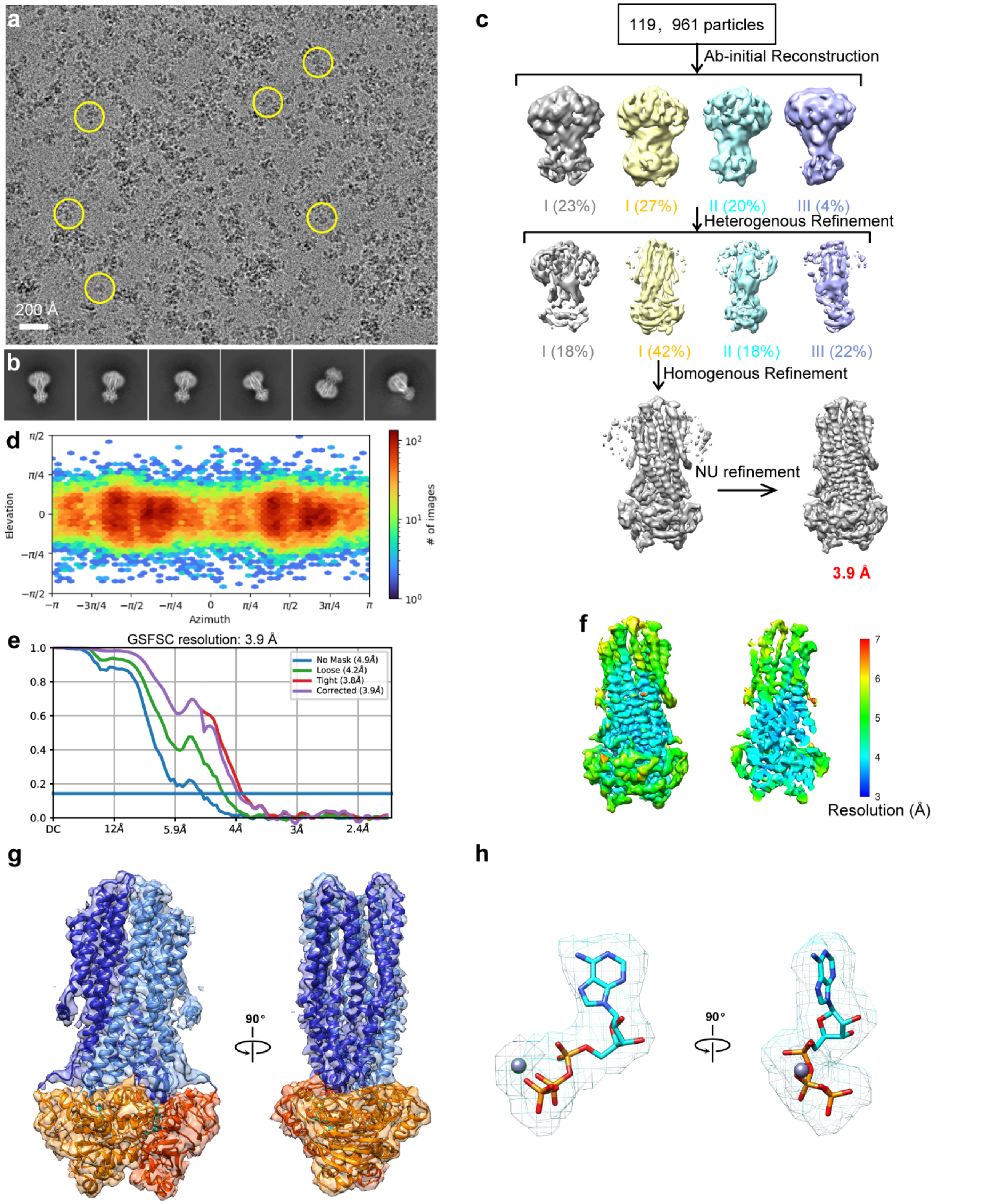

Supplementary Figure 16. Single-particle cryo-EM analysis of ATP-Zn^{2+} bound ComA. a, Representative cryo-EM image with several particles marked by circles. **b,** 2D averages of cryo-EM particle images. The box dimension is 280 Å. **c,** Image processing flowchart. The final maps with overall resolutions are indicated in red. **d,** The angular distribution of the cryo-EM particles included in the final 3D reconstruction. **e,** The Fourier shell correlation (FSC) curve: gold standard FSC between two half data maps with indicated resolution at FSC=0.143 (FSC corrected applied). **f,** The surface cryo-EM map filtered to the estimated overall resolution and colored according to local resolution. **g,** Superposition of cryo-EM density and the model fo ATP-Zn^{2+} bound ComA. **h,** Superposition of cryo-EM density and the model for ATP and Zn^{2+}. The contour level for EM density map in Chimera is 0.115.

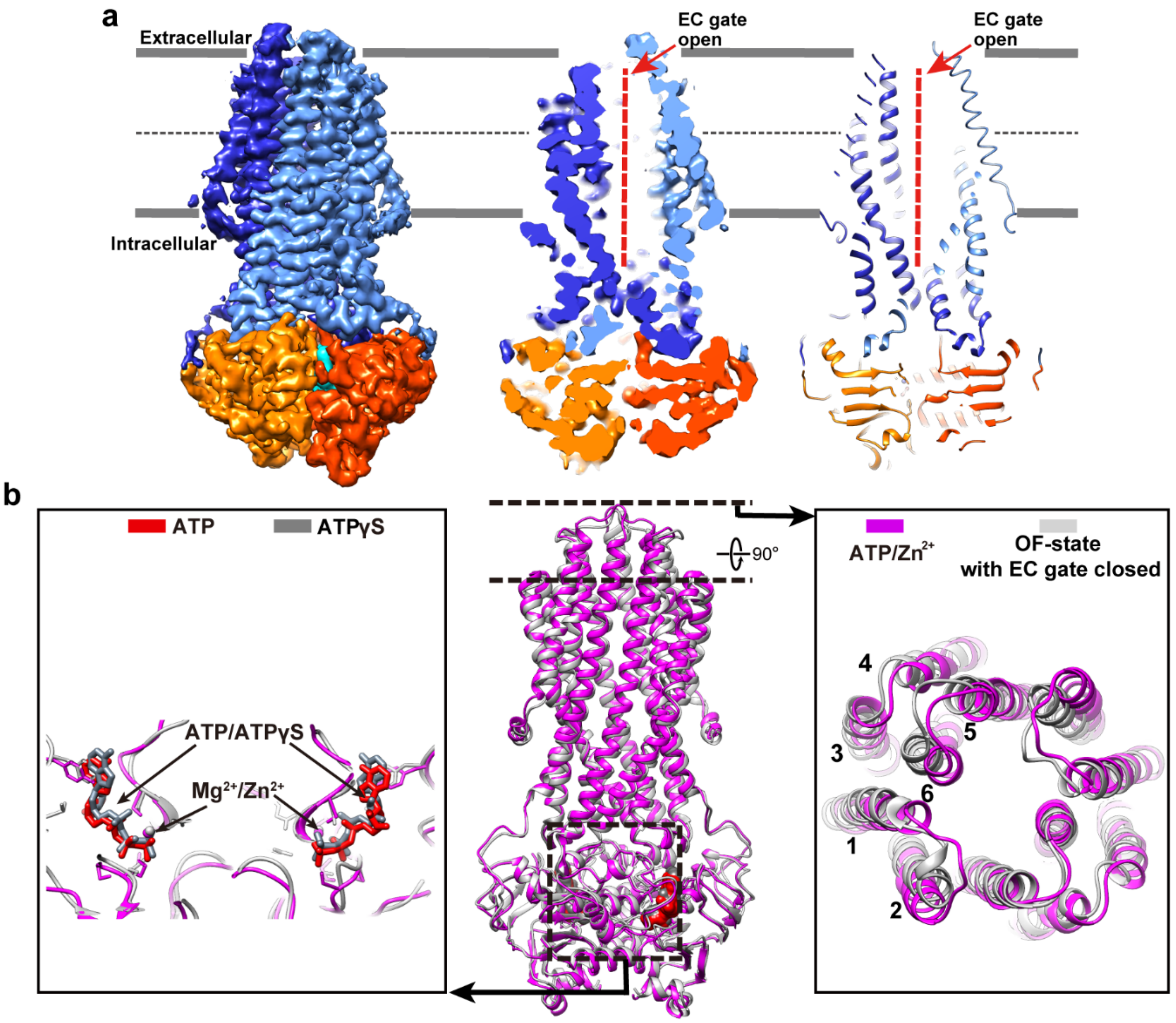

Supplementary Figure 17. Structure comparason between the ATP-Zn^{2+}-bound ComA and the OF-state with the EC Gate Open. a, Overall and sliced views of the ATP-Zn^{2+}-bound ComA model, depicting the open EC gate. The contour level for the EM map is set to 0.019 in Chimera. **b,** Superposition of the ATP-Zn^{2+}-bound ComA structure (in magenta, with ATP-Zn^{2+} colored in red) and the OF-state structure with the EC gate open (in grey, with ATPγS -Mg^{2+} colored in dim grey). The middle panel displays the overall alignment, while the left and right panels provide close-up views of the nucleotide-binding sites and the EC gate, respectively.

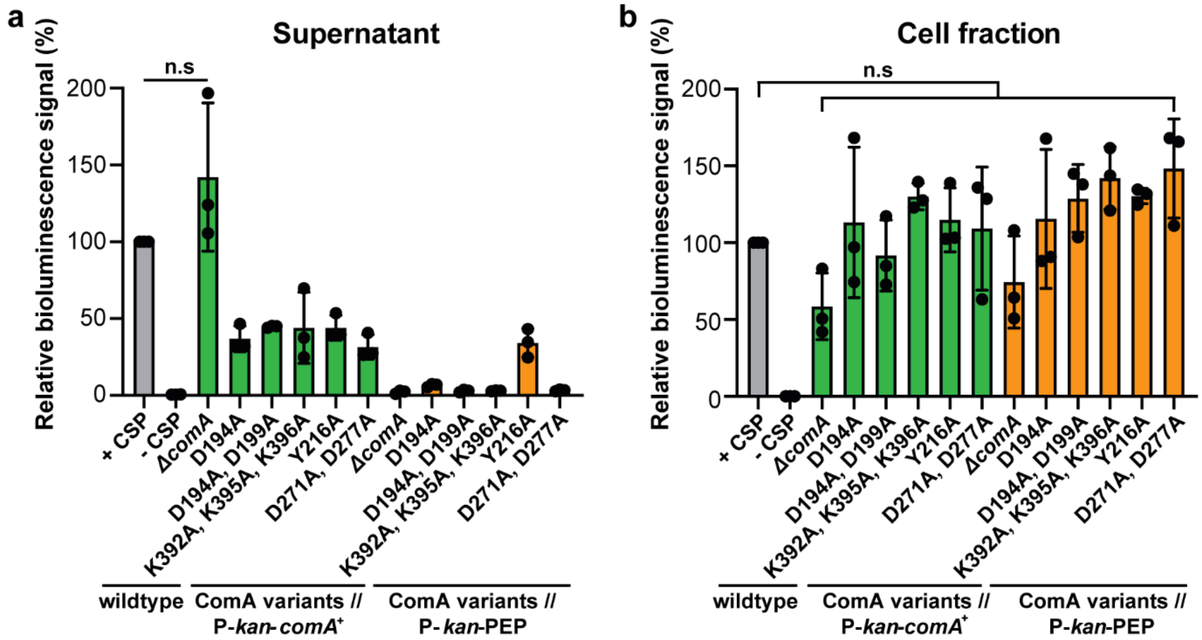

Supplementary Figure 18. Complementation of key residues identified for ComA function. a,b Defective ComA mutants were complemented with full length ComA (Green) and the PEP domain of ComA (Orange). *P* values were computed from three biological replicates using the Student's t test. Individual data points are presented as circles whereas standard deviation (SD) are shown as error bars (n=3 for all samples.)

Supplementary Table 1. List of cross-links identified for ComA ATP-free and ComA ATP-present samples.

Protein-state	Crosslink Residue 1	Crosslink Residue 2	Mapped Cα-Cα distance (Å)	Residue1 Domain	Residue2 Domain
ComA ATP-free	5	487	51.3		
ComA ATP-free	5	523	55.4		
ComA ATP-free	43	121	31.9		
ComA ATP-free	43	377	31.8	PEP	IC gate
ComA ATP-free	43	380	34.2	PEP	IC gate
ComA ATP-free	121	121	0		
ComA ATP-free	121	372	45.2	PEP	IC gate
ComA ATP-free	121	481	65		
ComA ATP-free	121	487	53.2		
ComA ATP-free	121	521	51.4		
ComA ATP-free	121	523	54.2		
ComA ATP-free	121	524	53.4		
ComA ATP-free	121	694	52.3	PEP	IC gate
ComA ATP-free	163	377	22.6		
ComA ATP-free	311	379	41.5		
ComA ATP-free	359	684	28.4		
ComA ATP-free	369	379	17.6		
ComA ATP-free	369	380	20.9		
ComA ATP-free	369	487	41.4		
ComA ATP-free	372	380	16.2		
ComA ATP-free	372	694	50.8		
ComA ATP-free	379	487	56.6		
ComA ATP-free	471	481	21.5		
ComA ATP-free	471	491	17.3		
ComA ATP-free	472	485	20.2		
ComA ATP-free	481	491	23.8		
ComA ATP-free	487	487	0		
ComA ATP-free	487	511	20.2		
ComA ATP-free	487	523	18.1		
ComA ATP-free	487	686	18.6		
ComA ATP-free	487	694	12.3		
ComA ATP-free	491	523	13.4		
ComA ATP-free	491	684	27.7		
ComA ATP-free	491	686	23.7		
ComA ATP-free	491	694	11.9		
ComA ATP-free	492	686	26.1		
ComA ATP-free	523	686	16		

ComA ATP-free	684	694	21.5		
ComA ATP-free	686	686	0		
ComA ATP-free	686	694	16.6		
ComA ATP-present	5	487	51.3		
ComA ATP-present	43	121	31.9		
ComA ATP-present	121	121	0		
ComA ATP-present	121	372	45.2		
ComA ATP-present	121	379	39.1	PEP	ICgate
ComA ATP-present	121	523	54.2		
ComA ATP-present	121	694	52.3		
ComA ATP-present	369	379	17.6		
ComA ATP-present	372	380	16.2		
ComA ATP-present	380	473	45.7		
ComA ATP-present	471	473	6.2		
ComA ATP-present	472	492	20.7		
ComA ATP-present	473	485	20.2		
ComA ATP-present	487	487	0		
ComA ATP-present	491	684	27.7		
ComA ATP-present	491	694	11.9		
ComA ATP-present	492	686	26.1		
ComA ATP-present	523	684	18		
ComA ATP-present	523	686	16		

Supplementary Table 2. Cryo-EM data collection, refinement and validation statistics

	ATPγS-bound ComA (EC gate closed) (EMD-34712) (PDB 8HF4)	ATPγS-bound ComA (EC gate open) (EMD-34713) (PDB 8HF5)	ATP-bound ComA (E647Q) (EMD-34714) (PDB 8HF6)	CSP-bound ComA (EMD-34715) (PDB 8HF7)	ComA (C17A) at pre-cleavage state (EMD-34716)	ATP/Zn^{2+}-bound ComA (EMD-36882) (PDB 8K4B)	ATP-Mg^{2+}-bound ComA (E647Q) (EMD-36936) (PDB 8K7A)
**Data collection and processing**							
Magnification	300	300	300	300	300	300	300
Voltage (kV)							
Electron exposure (e–/Å^2)	42	42	42	42	42	35	42
Defocus range (μm)	-1.2 to 2.5	-1.2 to 2.5	-1.2 to 2.5	-1.2 to 2.5	-1.2 to 2.5	-1.2 to 2.5	-1.2 to 2.5
Pixel size (Å)	0.858	0.858	0.858	0.858	0.858	1.105	0.858
Symmetry imposed	C1	C1	C1	C2	C1	C1	C1
Initial particle images (no.)	1,762,872	1,762,872	1,021,065	996,472	469,862	119,961	143,081
Final particle images (no.)	108,592	135,756	84,967	114,803	25,560	50,384	47217
Map resolution (Å) FSC threshold	2.8	2.9	3.1	3.8	6.0	3.9	4.5
**Refinement**							
Initial model used (PDB code)	4S0F	4S0F	4S0F	4S0F	--	4S0F	4S0F
Model resolution (Å) FSC threshold	2.8	2.9	3.1	3.8	--	3.9	4.5
Map sharpening *B* factor (Å^2)	-158	-170	-136	-121	-50	-141	-118
**Model composition**							
Non-hydrogen atoms	8916	8918	8916		--	8918	8918
Protein residues				8978			
Ligands	1126	1126	1126		--	1126	1126
	AGC:2	MG:2	ATP:2	1152	--	ZN:2	MG:2
		AGS:2				ATP:2	ATP:2
***B* factors (Å^2)**							
Protein	111.99	114.53	97.17	212.99	--	180.55	219.75
Ligand	126.97	156.80	106.29	--	--	14.81	13.17
R.m.s. deviations							
Bond lengths (Å)	0.008(22)	0.011(28)	0.009(41)	0.006(7)	--	0.008(12)	0.007(18)
Bond angles (°)	0.730(11)	1.414(63)	1.048(52)	1.342(24)	--	1.445(14)	1.486(62)
Validation							
MolProbity score	1.70	2.25	2.18	1.88	--	1.91	1.78
Clashscore							
Poor rotamers	5.73	7.39	8.43	7.32	--	8.38	7.00
(%)	1.11	3.74	3.23	0.61	--	0.91	0.00
**Ramachandran plot**							
Favored (%)	94.92	93.94	95.10	92.22	--	92.78	94.56
Allowed (%)	4.42	5.44	4.63	6.99	--	7.04	5.26
Disallowed (%)	0.36	0.62	0.27	0.79	--	0.18	0.18

Supplementary Table 3. Bacterial strains used in this study

Strain	Relevant genotype[a,b]	Derivation	Selectable marker[c]	Source
IU1690	serotype 2 strain D39	-	None	[1]
IU1781	*rpsL1*	*rpsL1* x IU1690	Str^{R}	[2]
IU5150	Δ*cps ftsZ*-mCherry Δ*ftsX*::P$_{C}$-*aad9* // Δ*bgaA*::P$_{fcsK}$-*ftsX*$^{+}$	-	Spec^{R}	[3]
HMS0001	*rpsL1* Δ*cps2E*<>P-*kan- rpsL*$^{+}$	-	Kan^{R}, Str^{S}	[4]
NUS0029	*rpsL1* Δ*cps2E::P-spec- rpsL*$^{+}$	Δ*cps2E::P-spec-rpsL* x HMS0001	Spec^{R}, Str^{S}	This study
NUS3109	*rpsL1* Δ*blpA*::P-*erm*	Δ*blpA::p-erm* x IU1781	Erm^{R}	This study
NUS3117	*rpsL1* Δ*blpA*::P-*erm* Δ*comA*::P-*spec-rpsL*$^{+}$	Δ*comA*::P-*spec-rpsL*$^{+}$ x NUS3109	Erm^{R}, Spec^{R}, Str^{S}	This study
NUS3118	*rpsL1* Δ*blpA*::P-*erm* Δ*comC*::*P-spec-rpsL*$^{+}$	Δ*comC*::P-*spec-rpsL*$^{+}$ x NUS3109	Erm^{R}, Spec^{R}, Str^{S}	This study
NUS3137	*rpsL1* Δ*blpA*::P-*erm comC-HiBiT*	Δ*comC<>comC-HiBiT* x NUS3118	Erm^{R}, Str^{R}	This study
NUS3145	*rpsL1* Δ*blpA*::P-*erm comC(ΔR15-K17)*	Δ*comC<>comC(ΔR15-K17)* x NUS3118	Erm^{R}, Str^{R}	This study
NUS3146	*rpsL1* Δ*blpA*::P-*erm comC(R15A, K16A, K17A)*	Δ*comC<>comC(R15A, K16A, K17A)* x NUS3118	Erm^{R}, Str^{R}	This study
NUS3148	*rpsL1* Δ*blpA*::P-*erm comA(D199A)*	Δ*comA<>comA(D199A)* x NUS3117	Erm^{R}, Str^{R}	This study
NUS3157	*rpsL1* Δ*blpA*::P-*erm comC*::HiBiT Δ*comA*::P-*spec-rpsL*$^{+}$	Δ*comA*::P-*spec-rpsL*$^{+}$ x NUS3137	Erm^{R}, Spec^{R}, Str^{S}	This study
NUS3166	*rpsL1* Δ*blpA*::P-*erm comC(D10N)-HiBiT*	Δ*comC<>comC(D10N)-HiBiT* x NUS3118	Erm^{R}, Str^{R}	This study
NUS3167	*rpsL1* Δ*blpA*::P-*erm comC(ΔR15-K17)-HiBiT*	Δ*comC<>comC(ΔR15-K17)-HiBiT* x NUS3118	Erm^{R}, Str^{R}	This study
NUS3168	*rpsL1* Δ*blpA*::P-*erm comC(R15A, K16A, K17A)-HiBiT*	Δ*comC<>comC(R15A, K16A, K17A)-HiBiT* x NUS3118	Erm^{R}, Str^{R}	This study
NUS3169	*rpsL1* Δ*blpA*::P-*erm comC(E1Q)-HiBiT*	Δ*comC<>comC(E1Q)-HiBiT* x NUS3118	Erm^{R}, Str^{R}	This study
NUS3170	*rpsL1* Δ*blpA*::P-*erm comC(E1R)-HiBiT*	Δ*comC<>comC(E1R)-HiBiT* x NUS3118	Erm^{R}, Str^{R}	This study
NUS3171	*rpsL1* Δ*blpA*::P-*erm comC(R3A)-HiBiT*	Δ*comC<>comC(R3A)-HiBiT* x NUS3118	Erm^{R}, Str^{R}	This study
NUS3178	*rpsL1* Δ*blpA*::P-*erm comA(D199A) comC-HiBiT*	Δ*comA<>comA(D199A)* x NUS3157	Erm^{R}, Str^{R}	This study
NUS3179	*rpsL1* Δ*blpA*::P-*erm comA(D194A) comC-HiBiT*	Δ*comA<>comA(D194A)* x NUS3157	Erm^{R}, Str^{R}	This study
NUS3179	*rpsL1* Δ*blpA*::P-*erm comA(D194A) comC-HiBiT*	Δ*comA<>comA(D194A)* x NUS3157	Erm^{R}, Str^{R}	This study
NUS3180	*rpsL1* Δ*blpA*::P-*erm comA(D194A, D199A) comC-HiBiT*	Δ*comA<>comA(D194A, D199A)* x NUS3157	Erm^{R}, Str^{R}	This study
NUS3181	*rpsL1* Δ*blpA*::P-*erm comA(K392A, K395A, K396A)*	Δ*comA<>comA(K392A, K395A, K396A)* x NUS3157	Erm^{R}, Str^{R}	This study

	comC-HiBiT			
NUS3182	*rpsL1* Δ*blpA*::P-*erm comA(Y216A) comC-HiBiT*	Δ*comA<>comA(Y216A)* x NUS3157	Erm^R, Str^R	This study
NUS3183	*rpsL1* Δ*blpA*::P-*erm comA(Y433A) comC-HiBiT*	Δ*comA<>comA(Y433A)* x NUS3157	Erm^R, Str^R	This study
NUS3184	*rpsL1* Δ*blpA*::P-*erm comA(N436A) comC-HiBiT*	Δ*comA<>comA(N436A)* x NUS3157	Erm^R, Str^R	This study
NUS3185	*rpsL1* Δ*blpA*::P-*erm comA(E439A) comC-HiBiT*	Δ*comA<>comA(E439A)* x NUS3157	Erm^R, Str^R	This study
NUS3186	*rpsL1* Δ*blpA*::P-*erm comA(H399A) comC-HiBiT*	Δ*comA<>comA(H399A)* x NUS3157	Erm^R, Str^R	This study
NUS3187	*rpsL1* Δ*blpA*::P-*erm comA(D271A, D277A) comC-HiBiT*	Δ*comA<>comA(D271A, D277A)* x NUS3157	Erm^R, Str^R	This study
NUS4464	*rpsL1* Δ*blpA*::P-*erm comC::HiBiT* Δ*comA*::P-*spec-rpsL* Δ*CEP*::P-*kan-comA*	Δ*CEP*-P-*kan-comA* x NUS3157	Spec^R, Kan^R, Erm^R	This study
NUS4465	*rpsL1* Δ*blpA*::P-*erm comA(D194A) comC-HiBiT* Δ*CEP*::P-*kan-comA*	Δ*CEP*-P-*kan-comA* x NUS3179	Kan^R, Erm^R, Str^R	This study
NUS4466	*rpsL1* Δ*blpA*::P-*erm comA(D194A, D199A) comC-HiBiT* Δ*CEP*::P-*kan-comA*	Δ*CEP*-P-*kan-comA* x NUS3180	Kan^R, Erm^R, Str^R	This study
NUS4467	*rpsL1* Δ*blpA*::P-*erm comA(K392A, K395A, K396A) comC-HiBiT* Δ*CEP*::P-*kan-comA*	Δ*CEP*-P-*kan-comA* x NUS3181	Kan^R, Erm^R, Str^R	This study
NUS4468	*rpsL1* Δ*blpA*::P-*erm comA(Y216A) comC-HiBiT* Δ*CEP*::P-*kan-comA*	Δ*CEP*-P-*kan-comA* x NUS3182	Kan^R, Erm^R, Str^R	This study
NUS4469	*rpsL1* Δ*blpA*::P-*erm comA(D271A, D277A) comC-HiBiT* Δ*CEP*::P-*kan-comA*	Δ*CEP*-P-*kan-comA* x NUS3187	Kan^R, Erm^R, Str^R	This study
NUS4470	*rpsL1* Δ*blpA*::P-*erm comC::HiBiT* Δ*comA*::P-*spec-rpsL* Δ*CEP*::P-*kan-comA(PEP)*	Δ*CEP*::P-*kan-comA*(*PEP*) x NUS3157	Spec^R, Kan^R, Erm^R	This study
NUS4471	*rpsL1* Δ*blpA*::P-*erm comA(D194A) comC-HiBiT* Δ*CEP*::P-*kan-comA(PEP)*	Δ*CEP*::P-*kan-comA*(*PEP*) x NUS3179	Kan^R, Erm^R, Str^R	This study
NUS4472	*rpsL1* Δ*blpA*::P-*erm comA(D194A, D199A) comC-HiBiT* Δ*CEP*::P-*kan-comA(PEP)*	Δ*CEP*::P-*kan-comA*(*PEP*) x NUS3180	Kan^R, Erm^R, Str^R	This study
NUS4473	*rpsL1* Δ*blpA*::P-*erm comA(K392A, K395A, K396A) comC-HiBiT* Δ*CEP*::P-*kan-comA(PEP)*	Δ*CEP*::P-*kan-comA*(*PEP*) x NUS3181	Kan^R, Erm^R, Str^R	This study
NUS4474	*rpsL1* Δ*blpA*::P-*erm comA(Y216A) comC-HiBiT* Δ*CEP*::P-*kan-comA(PEP)*	Δ*CEP*::P-*kan-comA*(*PEP*) x NUS3182	Kan^R, Erm^R, Str^R	This study
NUS4475	*rpsL1* Δ*blpA*::P-*erm comA(D271A, D277A) comC-*	Δ*CEP*::P-*kan-comA*(*PEP*) x NUS3187	Kan^R, Erm^R, Str^R	This study

	HiBiT Δ*CEP::*P-*kan-comA(PEP)*			

[a] Strains were constructed by transformation of amplicons (left of “x”) into the indicated recipient strain (right of “x”) as described in *Experimental procedures*. <> or :: indicates the exact replacement of a reading frame or insertion, respectively. Primers for constructing the strains are listed in Table S3.

[b] “P” refers to the constitutive synthetic promoter that drives the erythromycin resistant gene and the Janus cassette (*2*).

[c] Selectable markers: Erm , erythromycin; Str , streptomycin; Spec, spectinomycin.

Supplementary Table 4. Oligonucleotides used in this study

Primer	Sequence (5' to 3')	Template	Amplicon
For construction of Δ*blpA::p-erm*			
O2407	CCAAATGTTGCAAAGACGAAGA	D39 gDNA	*blpA* 5’
O2408	CATTATCCATTAAAAATCAAACGGATCCTAAGGAA<br>CAAATGTACGTTTATAAGAAGTC		
O1	TAGGATCCGTTTGATTTTTAATGGATAATG	P-*erm* cassette[a]	P-*erm*
O2	GGGCCCCTTTCCTTATGCTTTTG		
O2409	GTCCAAAAGCATAAGGAAAGGGGCCCGGCTTCTA<br>CCATCATCTATTCAATAAATAAGG	D39 gDNA	*blpA* 3’
O2410	GCAATTTCTGTACCAGTTGGAAT		
For construction of Δ*cps2E::P-spec-rpsL*$^+$			
P64	ACCAGCTACGACTCCTTCTTCT	HMS0001	*cps2E* 5’
P100	CACATTACCATTAAAAATCAAACGGGGGATCTTATT<br>ATTTCCTTCCTCTTTTCTACAG		
P101	GATCCCCCGTTTGATTTTTAATGGTAATGTG	IU5150	P-*spec*
P102	GATCCAATTTTTTTATAATTTTTTTAAT		
P99	ATTAAAAAAATTATAAAAAAATTGGATCTAGTACCTA<br>G<br>AATTCACCAAAAATAAAAAAAC	HMS0001	*rpsL*$^+$-*cps2E* 3’
P65	TCGTCCCACCACTAGATAATAGCC		
For construction of Δ*comA::P-spec-rpsL*$^+$			
O2418	AAGTTGTCTAGCTAAGTCATAGTAAGG	D39 gDNA	*comA* 5’
O2419	CATTATCCATTAAAAATCAAACGGATCCTACGGAC<br>GATAGTGACGTTTCCCAAATTTC		
O1	TAGGATCCGTTTGATTTTTAATGGATAATG	P-*spec*-*rpsL*$^+$ cassette[a]	P-*spec-rpsL*$^+$
O2	GGGCCCCTTTCCTTATGCTTTTG		
O2420	CCAAAAGCATAAGGAAAGGGGCCCGGCTTTTACG<br>CCCATTTGGTCAATAGCTAGAAAG	D39 gDNA	*comA* 3’
O2421	AAGATTTCCCTGTACCTTCTTACC		
For construction of Δ*comC::P-spec-rpsL*$^+$			
O2401	ACTACCCAAGGCTCCACTAA	D39 gDNA	*comC* 5’
O2413	CATTATCCATTAAAAATCAAACGGATCCTAAATAAA<br>ATCTCCTAAAATGTTTTTTCTTG		
O1	TAGGATCCGTTTGATTTTTAATGGATAATG	P-*spec*-*rpsL*$^+$ cassette[a]	P-*spec-rpsL*$^+$
O2	GGGCCCCTTTCCTTATGCTTTTG		
O2414	CAAAAGCATAAGGAAAGGGGCCCTGAAATAAGGG<br>GAAAGAGTAATGGATTTATTTGG	D39 gDNA	*comC* 3’
O2404	CTCTACACCATTATTTCGAGCATAGA		
For construction of Δ*comC<>comC-HiBiT*			
O2401	ACTACCCAAGGCTCCACTAA	D39 gDNA	*comC-HiBiT*
O2402	GCTGATTTTTTTGAAAAGACGCCATCCTGAAACCT<br>TTTTTCTTTGTAAAATAAAATCACG		
O2403	GTTTCAGGATGGCGTCTTTTCAAAAAAATCAGCTA<br>ATGAAATAAGGGGAAAGAGTAATGG	D39 gDNA	*comC-HiBiT*

O2404	CTCTACACCATTATTTCGAGCATAGA		
For construction of *ΔcomC<>comC(ΔR15-K17)*			
O2401	ACTACCCAAGGCTCCACTAA	D39 gDNA	*comC* (*ΔR15-K17*) 5’
O2450	TTCCCCTTATTTCATTATTGTAAAATAAAATCAC		
O2451	GTGATTTTATTTTACAATAATGAAATAAGGGGAA	D39 gDNA	*comC* (*ΔR15-K17*) 3’
O2404	CTCTACACCATTATTTCGAGCATAGA		
For construction of *ΔcomC<>comC(R15A, K16A, K17A)*			
O2401	ACTACCCAAGGCTCCACTAA	D39 gDNA	*comC* (*R15A, K16A, K17A*) 5’
O2448	TCCCCTTATTTCATTACGCTGCTGCTTGTAAAATAAAATCAC		
O2449	GTGATTTTATTTTACAAGCAGCAGCGTAATGAAATAAGGGGA	D39 gDNA	*comC* (*R15A, K16A, K17A*) 3’
O2404	CTCTACACCATTATTTCGAGCATAGA		
For construction of *ΔcomC<>comC(E1Q)-HiBiT*			
O2401	ACTACCCAAGGCTCCACTAA	NUS3137	*comC(E1Q)-HiBiT* 5’
O2442	TTTTGACAACCTCATCTGCCCACCTTTAATCTTTT		
O2443	AAAAGATTAAAGGTGGGCAGATGAGGTTGTCAAAA	NUS3137	*comC(E1Q)-HiBiT* 3’
O2404	CTCTACACCATTATTTCGAGCATAGA		
For construction of *ΔcomC<>comC(E1R)-HiBiT*			
O2401	ACTACCCAAGGCTCCACTAA	NUS3137	*comC(E1R)-HiBiT* 5’
O2444	ATTTTGACAACCTCATCCGCCCACCTTTAATCTTTT		
O2445	AAAAGATTAAAGGTGGGCGGATGAGGTTGTCAAAAT	NUS3137	*comC(E1R)-HiBiT* 3’
O2404	CTCTACACCATTATTTCGAGCATAGA		
For construction of *ΔcomC<>comC(D10N)-HiBiT*			
O2401	ACTACCCAAGGCTCCACTAA	NUS3137	*comC(D10N)-HiBiT* 5’
O2440	TCTTTGTAAAATAAAATTACGGAAGAATTTTGACA		
O2441	TGTCAAAATTCTTCCGTAATTTTATTTTACAAAGA	NUS3137	*comC(D10N)-HiBiT* 3’
O2404	CTCTACACCATTATTTCGAGCATAGA		
For construction of *ΔcomC<>comC(R3A)-HiBiT*			
O2401	ACTACCCAAGGCTCCACTAA	NUS3137	*comC(R3A)-HiBiT* 5’
O2446	GGAAGAATTTTGACAACGCCATCTCCCCACCTTTAA		
O2447	TTAAAGGTGGGGAGATGGCGTTGTCAAAATTCTTCC	NUS3137	*comC(R3A)-HiBiT* 3’
O2404	CTCTACACCATTATTTCGAGCATAGA		
For construction of *ΔcomC<>comC(ΔR15-K17)-HiBiT*			
O2401	ACTACCCAAGGCTCCACTAA	NUS3145	*comC(ΔR15-K17)-HiBiT* 5’
O2613	GCTGATTTTTTTGAAAAGACGCCATCCTGAAACTTGTAAAATAAAATCACGGAAGAATTT		
O2614	GTTTCAGGATGGCGTCTTTTCAAAAAAATCAGCTAATGAAATAAGGGGAAAGAGTAATGG	NUS3145	*comC(ΔR15-K17)-HiBiT* 3’
O2404	CTCTACACCATTATTTCGAGCATAGA		
For construction of *ΔcomC<>comC(R15A, K16A, K17A)-HiBiT*			
O2401	ACTACCCAAGGCTCCACTAA	NUS3146	

O2615	GCTGATTTTTTTGAAAAGACGCCATCCTGAAACCG CTGCTGCTTGTAAAATAAAATCACG		*comC(R15A, K16A, K17A)-HiBiT* 5’
O2614	GTTTCAGGATGGCGTCTTTTCAAAAAAATCAGCTA ATGAAATAAGGGGAAAGAGTAATGG	NUS3146	*comC(R15A, K16A, K17A)-HiBiT* 3’
O2404	CTCTACACCATTATTTCGAGCATAGA		
For construction of Δ*comA<>comA(D199A)*			
O2418	AAGTTGTCTAGCTAAGTCATAGTAAGG	D39 gDNA	*comA*(*D199A*) 5’
O2424	GTGTCGAACGCATCTGAGCTGGCACATAGGTATC A		
O2425	TGATACCTATGTGCCAGCTCAGATGCGTTCGACAC	D39 gDNA	*comA*(*D199A*) 3’
O2421	AAGATTTCCCTGTACCTTCTTACC		
For construction of Δ*comA<>comA(D194A)*			
O2418	AAGTTGTCTAGCTAAGTCATAGTAAGG	D39 gDNA	*comA*(*D194A*) 5’
O2422	GATCTGGCACATAGGTAGCAATGATAGACTGCAGA		
O2423	TCTGCAGTCTATCATTGCTACCTATGTGCCAGATC	D39 gDNA	*comA*(*D194A*) 3’
O2421	AAGATTTCCCTGTACCTTCTTACC		
For construction of Δ*comA<>comA(D194A, D199A)*			
O2418	AAGTTGTCTAGCTAAGTCATAGTAAGG	NUS3148	*comA*(*D194A, D199A*) 5’
O2422	GATCTGGCACATAGGTAGCAATGATAGACTGCAGA		
O2423	TCTGCAGTCTATCATTGCTACCTATGTGCCAGATC	NUS3148	*comA*(*D194A, D199A*) 3’
O2421	AAGATTTCCCTGTACCTTCTTACC		
For construction of Δ*comA<>comA(K392A, K395A, K396A)*			
O2418	AAGTTGTCTAGCTAAGTCATAGTAAGG	D39 gDNA	*comA*(*K392A,K395A, K396A*) 5’
O2432	GCAAGAGATGGGCAACCGCTGCCAGAGCCGCTTG CTGACTCTCTGCTC		
O2433	GAGCAGAGAGTCAGCAAGCGGCTCTGGCAGCGG TTGCCCATCTCTTGC	D39 gDNA	*comA*(*K392A,K395A, K396A*) 3’
O2421	AAGATTTCCCTGTACCTTCTTACC		
For construction of Δ*comA<>comA(Y216A)*			
O2418	AAGTTGTCTAGCTAAGTCATAGTAAGG	D39 gDNA	*comA*(*Y216A*) 5’
O2436	AGATTTGCTGGAGGATGGCGACGATGACTAGCCC AA		
O2437	TTGGGCTAGTCATCGTCGCCATCCTCCAGCAAATC T	D39 gDNA	*comA*(*Y216A*) 3’
O2421	AAGATTTCCCTGTACCTTCTTACC		
For construction of Δ*comA<>comA(Y433A)*			
O2418	AAGTTGTCTAGCTAAGTCATAGTAAGG	D39 gDNA	*comA*(*Y433A*) 5’
O2438	CCAAAGGATTGGTAAAGGCAACCAGCAAGGTATTA T		
O2439	ATAATACCTTGCTGGTTGCCTTTACCAATCCTTTGG	D39 gDNA	*comA*(*Y433A*) 3’
O2421	AAGATTTCCCTGTACCTTCTTACC		
For construction of Δ*comA<>comA(N436A)*			
O2418	AAGTTGTCTAGCTAAGTCATAGTAAGG	D39 gDNA	*comA*(*N436A*) 5’
O2434	TGATATTTTCCAAAGGAGCGGTAAAGTAAACCAGC A		

O2435	TGCTGGTTTACTTTACCGCTCCTTTGGAAAATATCA	D39 gDNA	*comA*(*N436A*) 3’
O2421	AAGATTTCCCTGTACCTTCTTACC		
For construction of Δ*comA<>comA(E439A)*			
O2418	AAGTTGTCTAGCTAAGTCATAGTAAGG	D39 gDNA	*comA*(E439A) 5’
O2428	GCAGATTGATGATATTTGCCAAAGGATTGGTAAAG		
O2429	CTTTACCAATCCTTTGGCAAATATCATCAATCTGC	D39 gDNA	*comA*(E439A) 3’
O2421	AAGATTTCCCTGTACCTTCTTACC		
For construction of Δ*comA<>comA(H399A)*			
O2418	AAGTTGTCTAGCTAAGTCATAGTAAGG	D39 gDNA	*comA*(*H399A*) 5’
O2430	CGACATTAAGCAAGAGAGCGGCAACCTTTTTCAGA G		
O2431	CTCTGAAAAAGGTTGCCGCTCTCTTGCTTAATGTC G	D39 gDNA	*comA*(*H399A*) 3’
O2421	AAGATTTCCCTGTACCTTCTTACC		
For construction of Δ*comA<>comA(D271A, D277A)*			
O2418	AAGTTGTCTAGCTAAGTCATAGTAAGG	D39 gDNA	*comA*(*D271A, D277A*) 5’
O2426	TGGTCGAAGCCAGCGCAGCGATGATACTGTTAGC AGCTGTAAAACGAGACACG		
O2427	CGTGTCTCGTTTTACAGCTGCTAACAGTATCATCG CTGCGCTGGCTTCGACCA	D39 gDNA	*comA*(*D271A, D277A*) 3’
O2421	AAGATTTCCCTGTACCTTCTTACC		
For construction of Δ*CEP'*-P-*kan*-*comA*-*CEP'*			
O2093	GCTGACTAGGAGGAAGGAAATG	S208	*CEP'-P-kan*
O2680	CGATAGTGACGTTTCCCAAATTTCATCTACATTCTC CTGTGTTTTTTTATTTTTGG		
O2506	ATGAAATTTGGGAAACGTCACTATC	D39 gDNA	*comA*
O2507	CTAGCTATTGACCAAATGGGCGTAA		
O4268	TTACGCCCATTTGGTCAATAGCTAGGGAAAGGGG CCCGTCGCTTTTCATTATAGG	D39	*CEP'*
O2094	TCTTTACCTCCAATTGCCTGAA		
For construction of Δ*CEP'*-P-*kan*-*comA*(*PEP*)-*CEP'*			
O2093	GCTGACTAGGAGGAAGGAAATG	S208	*CEP'-P-kan*
O2680	CGATAGTGACGTTTCCCAAATTTCATCTACATTCTC CTGTGTTTTTTTATTTTTGG		
O2506	ATGAAATTTGGGAAACGTCACTATC	D39 gDNA	*comA(PEP)* domain
O4269	CTACAGACCATTTTTTTGTTCCTTA		
O4270	CATAAGGAACAAAAAAATGGTCTGTAGGGAAAGG GGCCCGTCGCTTTTCATTATAGG	D39	*CEP'*
O2094	TCTTTACCTCCAATTGCCTGAA		

[a] P-*erm* cassette was kindly provided by the laboratory of Malcolm Winkler.

References

1 Lanie, J. A. *et al.* Genome sequence of Avery's virulent serotype 2 strain D39 of Streptococcus pneumoniae and comparison with that of unencapsulated laboratory strain R6. *J Bacteriol* **189**, 38-51, doi:10.1128/JB.01148-06 (2007).

2 Kazmierczak, K. M., Wayne, K. J., Rechtsteiner, A. & Winkler, M. E. Roles of rel(Spn) in stringent response, global regulation and virulence of serotype 2 Streptococcus pneumoniae D39. *Mol Microbiol* **72**, 590-611, doi:10.1111/j.1365-2958.2009.06669.x (2009).

3 Sham, L. T., Barendt, S. M., Kopecky, K. E. & Winkler, M. E. Essential PcsB putative peptidoglycan hydrolase interacts with the essential FtsXSpn cell division protein in Streptococcus pneumoniae D39. *Proc Natl Acad Sci U S A* **108**, E1061-1069, doi:10.1073/pnas.1108323108 (2011).

4 Chua, W. Z. *et al.* High-Throughput Mutagenesis and Cross-Complementation Experiments Reveal Substrate Preference and Critical Residues of the Capsule Transporters in Streptococcus pneumoniae. *mBio* **12**, e0261521, doi:10.1128/mBio.02615-21 (2021).

Source Data

Supplementary Figure 1a

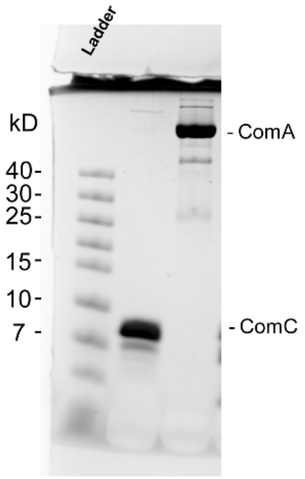

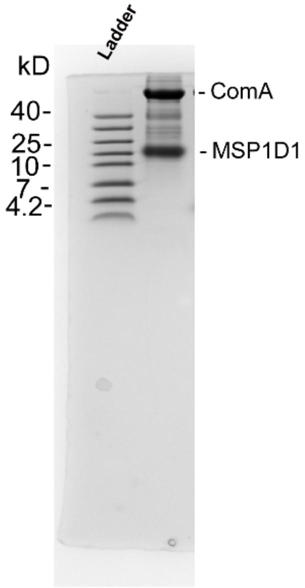

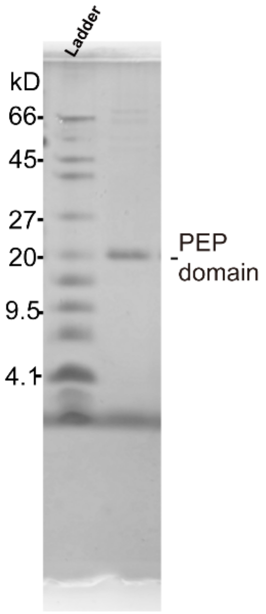

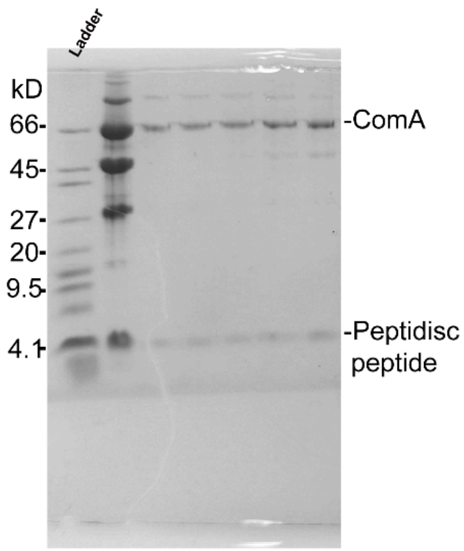

Supplementary Figure 1d

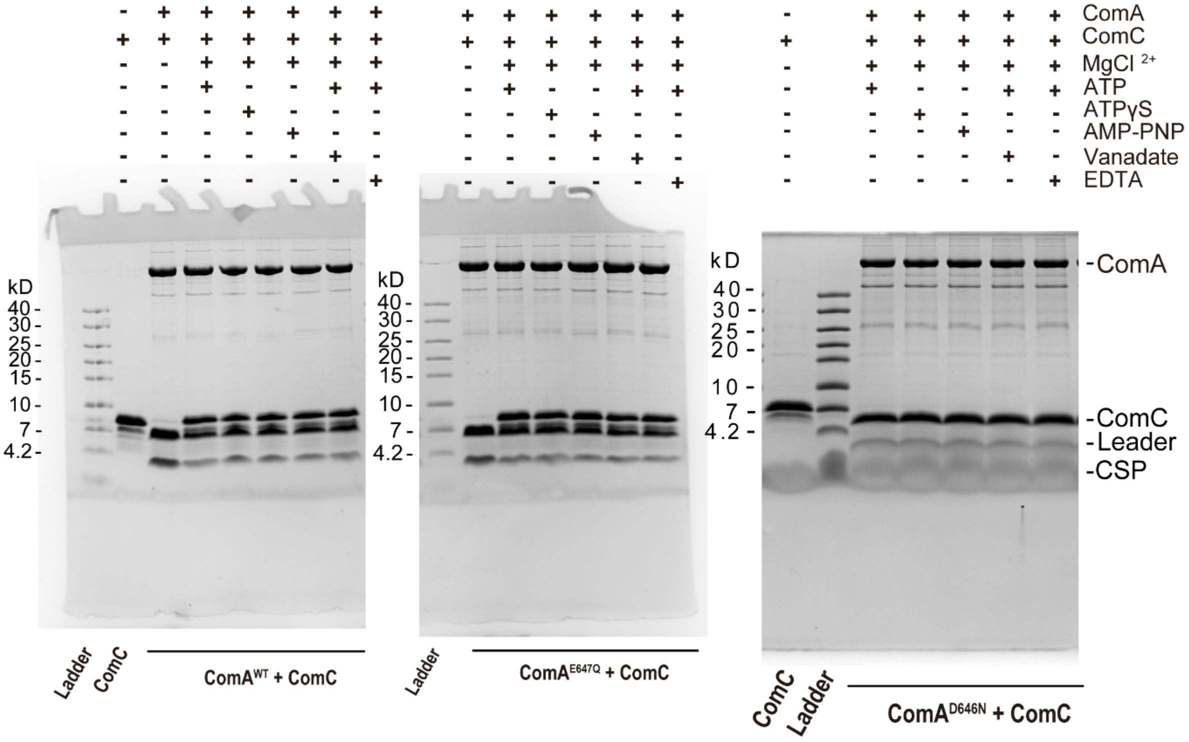

Supplementary Figure 1f

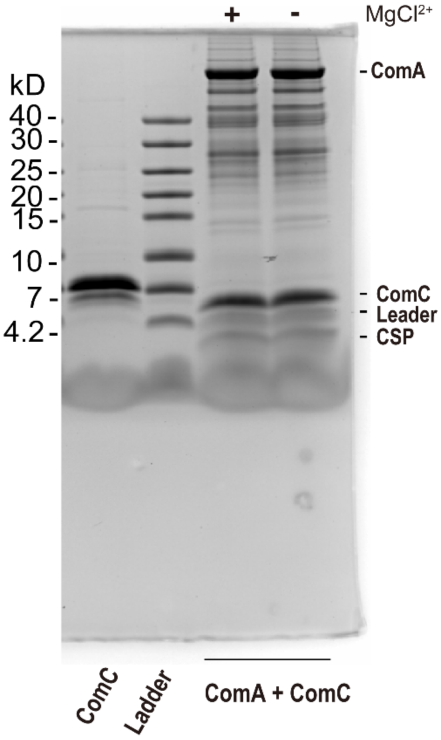

Supplement: Supplementary file 1 — Supplementary Information [file 41467_2023_42852_MOESM1_ESM.pdf]
