# Supplementary material for: Structural basis of peptide secretion for Quorum sensing by ComA
Source: Nat Commun. 2023 Nov 7;14:7178. doi: 10.1038/s41467-023-42852-9 (PMC10630487; doi:10.1038/s41467-023-42852-9)
Supplement: Supplementary file 3 — Reporting Summary [file 41467_2023_42852_MOESM3_ESM.pdf]

nature portfolio

Corresponding author(s): Luo Min

Last updated by author(s): Oct 12, 2023

Reporting Summary

Nature Portfolio wishes to improve the reproducibility of the work that we publish. This form provides structure for consistency and transparency in reporting. For further information on Nature Portfolio policies, see our Editorial Policies and the Editorial Policy Checklist.

Statistics

For all statistical analyses, confirm that the following items are present in the figure legend, table legend, main text, or Methods section.

n/a	Confirmed	
☐	☒	The exact sample size (*n*) for each experimental group/condition, given as a discrete number and unit of measurement
☐	☒	A statement on whether measurements were taken from distinct samples or whether the same sample was measured repeatedly
☒	☐	The statistical test(s) used AND whether they are one- or two-sided<br>*Only common tests should be described solely by name; describe more complex techniques in the Methods section.*
☐	☒	A description of all covariates tested
☒	☐	A description of any assumptions or corrections, such as tests of normality and adjustment for multiple comparisons
☐	☒	A full description of the statistical parameters including central tendency (e.g. means) or other basic estimates (e.g. regression coefficient) AND variation (e.g. standard deviation) or associated estimates of uncertainty (e.g. confidence intervals)
☐	☒	For null hypothesis testing, the test statistic (e.g. *F*, *t*, *r*) with confidence intervals, effect sizes, degrees of freedom and *P* value noted<br>*Give P values as exact values whenever suitable.*
☒	☐	For Bayesian analysis, information on the choice of priors and Markov chain Monte Carlo settings
☒	☐	For hierarchical and complex designs, identification of the appropriate level for tests and full reporting of outcomes
☒	☐	Estimates of effect sizes (e.g. Cohen's *d*, Pearson's *r*), indicating how they were calculated

Our web collection on statistics for biologists contains articles on many of the points above.

Software and code

Policy information about availability of computer code

Data collection	SerialEM version 4.0, UNICORN 7.0
Data analysis	CryoSPARC v3.3.2, MotionCor 2.0, CTFFIND v4.1, Coot v0.9.8.1, Phenix 1.2, UCFS Chimera v1.16

For manuscripts utilizing custom algorithms or software that are central to the research but not yet described in published literature, software must be made available to editors and reviewers. We strongly encourage code deposition in a community repository (e.g. GitHub). See the Nature Portfolio guidelines for submitting code & software for further information.

Data

Policy information about availability of data

All manuscripts must include a data availability statement. This statement should provide the following information, where applicable:

- Accession codes, unique identifiers, or web links for publicly available datasets
- A description of any restrictions on data availability
- For clinical datasets or third party data, please ensure that the statement adheres to our policy

Three-dimensional cryo-EM density maps of ComA in the presence and absence of bound CSP, nucleotides or divalent metal have been deposited in the Electron Microscopy Data Bank under accession codes: EMD-34712 [https://www.ebi.ac.uk/emdb/EMD-34712] (ATPγS-bound ComA, OF-state with EC gate closed); EMD-34713 [https://www.ebi.ac.uk/emdb/EMD-34713] (ATPγS-bound ComA, OF-state with EC gate open); EMD-34714 [https://www.ebi.ac.uk/emdb/EMD-34714] (ATP-bound ComA (E647Q), post-cleavage state); EMD-34715 [https://www.ebi.ac.uk/emdb/EMD-34715] (CSP-bound ComA); EMD-34716 [https://www.ebi.ac.uk/emdb/EMD-34716] (ComA C17A with ComC), EMD-36882 [https://www.ebi.ac.uk/emdb/EMD-36882] (ATP-Zn2+ bound ComA), EMD-36936 [https://

www.ebi.ac.uk/emdb/EMD-36936] (ATP-Mg2+ bound ComA (E647Q)). Atomic models have been deposited in the Protein Data Bank under accession codes 8HF4 [https://www.rcsb.org/structure/8HF4] (ATPγS-bound ComA, OF-state with EC gate closed), 8HF5 [https://www.rcsb.org/structure/8HF5] (ATPγS-bound ComA, OF-state with EC gate open), 8HF6 [https://www.rcsb.org/structure/8HF6] (ATP-bound ComA (E647Q), post-cleavage state), 8HF7 [https://www.rcsb.org/structure/8HF7] (CSP-bound ComA), 8K4B [https://www.rcsb.org/structure/8K4B] (ATP-Zn2+ bound ComA) and 8K7A [https://www.rcsb.org/structure/8K7A] (ATP-Mg2+ bound ComA (E647Q)). Raw mass spectrometry spectra and search data were uploaded to the jPost repository with the following accession numbers: JPST001916 (jPOST) and PXD038058 (ProteomeXchange).
For reviewers, raw mass spectrometry data can be accessed with access key 8953 at: https://repository.jpostdb.org/preview/1690629505636b3a9905bde. All data presented are available in the main text or the supplementary materials. The source data underlying Figures 1b, 1c, 1d, 1e, 1f, 4c, 4e, 4f, 6d, 7a-d and Supplementary Figure 1a, 1b, 1c, 1d, 1e, 1f, 6c, 9, 18 are provided as a Source Data file.

Research involving human participants, their data, or biological material

Policy information about studies with human participants or human data. See also policy information about sex, gender (identity/presentation), and sexual orientation and race, ethnicity and racism.

Reporting on sex and gender	not applicable
Reporting on race, ethnicity, or other socially relevant groupings	not applicable
Population characteristics	not applicable
Recruitment	not applicable
Ethics oversight	not applicable

Note that full information on the approval of the study protocol must also be provided in the manuscript.

Field-specific reporting

Please select the one below that is the best fit for your research. If you are not sure, read the appropriate sections before making your selection.

☒ Life sciences ☐ Behavioural & social sciences ☐ Ecological, evolutionary & environmental sciences

For a reference copy of the document with all sections, see nature.com/documents/nr-reporting-summary-flat.pdf

Life sciences study design

All studies must disclose on these points even when the disclosure is negative.

Sample size	No statistical methods were used to predetermine sample size. The number of particles used in structural determination was not predetermined. The purified protein complex were sufficient for the EM and biochemical analyses. The cryo images are also sufficient as many independently recorded images are acquired as part of cryo-EM data collection. The samples on the cryo-EM images are all chosen.
Data exclusions	For cryo-EM raw micrograph screening, we excluded images based on their quality and the presence of ice contamination. As for particle selection, we used criteria that depended on the quality of both the generated 2D class averages and the 3D map.
Replication	We conducted multiple rounds of structural refinement, consistently yielding the same density map. Each reported experiment was repeated at least three times, consistently producing consistent results.
Randomization	Randomization was not applicable since experimental samples were not grouped
Blinding	Investigators were not blinded to group allocation. It is not applicable to this study. This is because investigators need to design and analyze experiments based on samples, especially in cases related to structural biology.

Reporting for specific materials, systems and methods

We require information from authors about some types of materials, experimental systems and methods used in many studies. Here, indicate whether each material, system or method listed is relevant to your study. If you are not sure if a list item applies to your research, read the appropriate section before selecting a response.

Materials & experimental systems

n/a	Involved in the study
☒	☐ Antibodies
☒	☐ Eukaryotic cell lines
☒	☐ Palaeontology and archaeology
☒	☐ Animals and other organisms
☒	☐ Clinical data
☒	☐ Dual use research of concern
☒	☐ Plants

Methods

n/a	Involved in the study
☒	☐ ChIP-seq
☒	☐ Flow cytometry
☒	☐ MRI-based neuroimaging
